# Dual role of ANGPTL8 in promoting tumor cell proliferation and immune escape during hepatocarcinogenesis

**DOI:** 10.1038/s41389-023-00473-3

**Published:** 2023-05-15

**Authors:** Yujiu Gao, Yue Yuan, Shu Wen, Yanghui Chen, Zongli Zhang, Ying Feng, Bin Jiang, Shinan Ma, Rong Hu, Chen Fang, Xuzhi Ruan, Yahong Yuan, Xinggang Fang, Chao Luo, Zhongji Meng, Xiaoli Wang, Xingrong Guo

**Affiliations:** 1 Department of Critical Care Medicine, Hubei Key Laboratory of Embryonic Stem Cell Research, School of Basic Medical Sciences, Taihe Hospital, Hubei University of Medicine, 442000 Shiyan, China; 2 Department of Nephrology, Taihe Hospital, 442000 Shiyan, China; 3 Hubei Clinical Research Center for Umbilical Cord Blood Hematopoietic Stem Cells, Taihe Hospital, 442000 Shiyan, China; 4 College of Pharmacy, Hubei University of Medicine, 442000 Shiyan, China; 5 Department of Anesthesiology, Renmin Hospital of Wuhan University, 430060 Wuhan, China; 6 Institute of Pediatric Disease, Taihe Hospital, 442000 Shiyan, China; 7 Department of Hepatobiliary Pancreatic Surgery, Taihe Hospital, 442000 Shiyan, China; 8 Department of Infectious Diseases, Institute of Biomedical Research, Hubei Clinical Research Center for Precise Diagnosis and Treatment of Liver Cancer, Taihe Hospital, 442000 Shiyan, China

**Keywords:** Liver cancer, Autophagy, Immunosurveillance

## Abstract

The interplay between hepatocellular carcinoma (HCC) cells and the tumor microenvironment is essential for hepatocarcinogenesis, but their contributions to HCC development are incompletely understood. We assessed the role of ANGPTL8, a protein secreted by HCC cells, in hepatocarcinogenesis and the mechanisms through which ANGPTL8 mediates crosstalk between HCC cells and tumor-associated macrophages. Immunohistochemical, Western blotting, RNA-Seq, and flow cytometry analyses of ANGPTL8 were performed. A series of in vitro and in vivo experiments were conducted to reveal the role of ANGPTL8 in the progression of HCC. *ANGPTL8* expression was positively correlated with tumor malignancy in HCC, and high *ANGPTL8* expression was associated with poor overall survival (OS) and disease-free survival (DFS). ANGPTL8 promoted HCC cell proliferation in vitro and in vivo, and *ANGPTL8* KO inhibited the development of HCC in both DEN-induced and DEN-plus-CCL4-induced mouse HCC tumors. Mechanistically, the ANGPTL8–LILRB2/PIRB interaction promoted polarization of macrophages to the immunosuppressive M2 phenotype in macrophages and recruited immunosuppressive T cells. In hepatocytes, ANGPTL8-mediated stimulation of LILRB2/PIRB regulated the ROS/ERK pathway and upregulated autophagy, leading to the proliferation of HCC cells. Our data support the notion that ANGPTL8 has a dual role in promoting tumor cell proliferation and immune escape during hepatocarcinogenesis.

## Introduction

Primary liver cancer, which comprises eighty percent of hepatocellular carcinoma (HCC), is the sixth most common cancer worldwide [1]. Even with the development of advanced diagnostic and therapeutic strategies for HCC, the prognosis for patients with HCC remains poor owing to its complex and multifaceted molecular pathogenesis [2]. The mechanisms of carcinogen-mediated HCC are characterized by hepatocyte injury and death, which lead to abnormal cell proliferation [3]. In the context of a healthy immune system, aberrantly proliferating cells can be precisely targeted and eliminated by immune cells [4]. However, when the immune system is dysregulated, pathogenic cells are not efficiently cleared, thereby promoting the development of HCC and other tumors. Therefore, the host microenvironment is essential and in many cases even determinant during early tumorigenesis in the liver. However, the full complement of molecular signals and pathways that induce hepatocarcinogenesis are still incompletely understood.

Angiopoietin-like proteins (ANGPTLs) are a family of secreted glycoproteins that share common protein domains with an N-terminal coiled domain and a C-terminal fibrinogen-like domain. The exception to this is ANGPTL8 [5], which is a secreted protein that is predominantly expressed in the liver [6, 7], suggesting that ANGPTL8 may have functions different from those of other ANGPTLs. Although the expression and prognostic potential of ANGPTL8 in different cancers have been analyzed [8], these studies have primarily considered tumor molecular profiling data for their conclusions, and a few reports suggest that ANGPTL8 could promote the progression of renal cell carcinoma [9], but inhibit the development of breast invasive carcinoma and cholangiocarcinoma [10]. However, little is known regarding the function of ANGPTL8 in tumorigenesis. It is now evident that ANGPTL8 contributes to inflammatory diseases [11, 12], our previous research determined that ANGPTL8 is a proinflammatory cytokine secreted by hepatocytes into the liver microenvironment to regulate hepatic stellate cell activation, which in turn accelerates nonalcoholic fatty liver disease (NAFLD)-associated liver fibrogenesis [13]. Liver fibrosis, if not well controlled, can progress to HCC [14]. Although, we further demonstrated that *ANGPTL8* expression gradually increases as NAFLD progresses and is highest in patients with HCC [13], whether ANGPTL8 plays a role in hepatocarcinogenesis is unclear.

In this study, we show that HCC cells secrete ANGPTL8, which drives the proliferation of hepatocytes and induces an immunosuppressive microenvironment by promoting the polarization of macrophages to the M2 phenotype and recruiting immunosuppressive T cells. Our data support that ANGPTL8 has a dual role in promoting tumor cell proliferation and immune escape during hepatocarcinogenesis.

Our research indicates that ANGPTL8 may serve as an early diagnostic biomarker as well as a therapeutic target for HCC.

## Materials and methods

### Clinical samples and cell lines

The HCC specimens and the corresponding normal tissues used in this study were obtained from the First Affiliated Taihe Hospital of Hubei University of Medicine. The study was approved by the Ethics Committee of First Affiliated Taihe Hospital of Hubei University of Medicine.

HepG2, MHCC97H, LO2 and RAW264.7 cell lines were purchased from the China Type Culture Collection (CCTCC). All cells were routinely cultured in DMEM supplemented with 10% fetal bovine serum. The cells were incubated in a humidified incubator with 5% CO_2_ at 37 °C.

### DEN-induced HCC model

*ANGPTL8*^*−/−*^(*ANGPTL8* knockout, conventional knockout) mice were generated by CRISPR/Cas9-mediated genome engineering in C57BL/6J mice as previously described [15]. All animal care and experimental procedures were approved by the ethical review of the Laboratory Animal Management and Use Committee of Centers for Disease Control of Hubei Province. Male C57BL/6J wild-type (WT) and *ANGPTL8* knockout (KO) mice were used to establish DEN plus CCL4-induced and DEN only induced murine models of HCC. The DEN plus CCL4-induced model was induced by a single injection of 50 mg/kg DEN at 2 weeks of age, followed by administration of 1 μL/g carbon tetrachloride (CCL4) twice a week from the fourth week up to the 24th week. The DEN-induced model was intraperitoneally injected with 50 mg/kg DEN every 2 weeks 8 times at 2 weeks of age. The small animal ultrasound system was used to monitor tumor formation in the liver, and the mice were sacrificed at 40 weeks.

### Orthotopic HCC model

The model was established as we previously reported [16]. To monitor tumor growth in nude mice, 1 × 10^7^ MHCC97H-Luc and MHCC97H-Luc cells with *ANGPTL8* knockdown (KD) or overexpression (OE) were orthotopically injected into the livers of nude mice. The size of neoplasms from the implanted cells was detected by the IVS Spectrum system (Caliper Life Sciences) at 3, 10, 20, and 30 days. The bioluminescence signal was analyzed with software after the placement of a small region of interest. The mean light intensity was then measured within this region of interest. The survival rates of mice in each group were also recorded and calculated.

### Establishment of HCC cell lines with *ANGPTL8* KD or OE

HepG2 and MHCC97H cells with *ANGPTL8* KD or OE were established by a lentiviral-based CRISPR gene editing system (lentiCRISPR v2, Cyagen Biosciences) or lentiviruses overexpressing *ANGPTL8* (Cyagen Biosciences) according to the manufacturer’s instructions. The primer sequences are provided in Supplementary Table 1. The *ANGPTL8* expression level was confirmed by western blotting. Detailed methods are provided in the supplementary materials and methods.

### Isolation of primary mouse liver cells

Primary mouse hepatocytes (PMHs) and primary Kupffer cells (PKCs) were isolated by in situ retrograde perfusion of the liver with collagenase digestion medium [17], and the cell suspension was purified by discontinuous Percoll (Lablead, 17-0891-01) density gradient centrifugation as previously reported [18, 19].

### Western blotting

The membranes were incubated with antibodies against ANGPTL8 (ab180915, Abcam), CD133 (ab222782, Abcam), Beclin-1 (3495S, CST), ATG5 (AF2269, Beyotime), P62 (5114s, CST), LC3I/II (14600-1-AP, Proteintech), ERK1/2 (AF1051, Beyotime), p-ERK1/2 (4370s, CST), α-Tubulin (AF2827, Beyotime), β-tubulin (AF2835, Beyotime) and LILRB2 (A10135, ABclonal) at 4 °C overnight. The membranes were incubated with horseradish peroxidase (HRP)-conjugated secondary antibody at RT for 1 h. Then, the membranes were imaged with a gel imaging system (BIO-RAD, CA, USA). Primary antibodies used for western blotting are listed in Supplementary Table 2.

### RT-qPCR

Total RNA was extracted using TRIzol following the manufacturer’s recommendations and quantified by UV spectroscopy. To prepare RNA for PCR analysis, 2 μg of total RNA was converted to cDNA using a Fast Quant RT Kit with gDNase. RT-qPCR was performed using a standard SYBR Green PCR kit protocol on a Step One TM Real-Time PCR System. The expression of the genes was quantified by relative quantification using the comparative CT method. Primers for RT-qPCR are shown in Supplementary Table 3.

### RNA-Seq data analysis

RNA-seq of three pairs of WT and *ANGPTL8* KO mouse primary liver cancer tissue was performed by Novogene using Illumina X TEN. Clean data (6 GB) per sample were collected for RNA-seq. Differential expression analysis was performed to yield statistically significant features (p value < 0.05) between treatment groups using the Ballgown R package. Functional annotation analysis was carried out using Ingenuity Pathway AnalysisTM and CIBERSORT analysis.

### ANGPTL8 and PIR-B/Fc interaction assays

pMXs-IRES-green fluorescent protein (pMXsIG) vectors with PIRA1-6, PIRB, and CD3e were provided by Peixiang Lan, and the operation steps were performed according to previous reports with slight modifications [20]. These vectors were transfected into HEK-T293 cells and then incubated with 400 ng/mL rANGPTL8 or Ctrl IgG for 2 h. Luciferase activity was quantified using a luciferase-reporter-gene-assay system (Beyotime, China). As a control, an empty pMXsIG and CD3e vector was transfected into the cells.

### Statistical analysis

The data are expressed as the mean ± SD (standard deviation), and all data were analyzed with the SPSS 22.0 software. Data between two groups were compared by two-tailed Student’s *t* test. One-way ANOVA was used to compare multiple groups with a normal distribution. The least significant difference (LSD) or Tamhane’s T2 test was applied between groups for data meeting homogeneity of variance or showing heteroscedasticity. *p* < 0.05 was considered statistically significant.

## Results

### *ANGPTL8* is highly expressed in HCC cells and tissues

To determine whether the *ANGPTL8* expression level correlated with the development of HCC, we analyzed tissue sections of HCC of different stages and corresponding paracarcinoma tissue by immunohistochemistry and immunofluorescence. *ANGPTL8* expression was significantly increased in tumor tissue compared to normal tissue (Fig. S1A). Interestingly, we also noted that *ANGPTL8* expression was positively correlated with the expression of CD133, which was used as one of the indicators of the degree of malignancy (Fig. S1B). Next, we analyzed the TCGA-LIHC HCC cohort, which showed that patients with high *ANGPTL8* expression had significantly worse overall survival (OS) and disease-free survival (DFS) than patients with low *ANGPTL8* expression (Fig. S1C). Next, we evaluated *ANGPTL8* expression in HCC cell lines (MHCC97H, MHCC97 L, and HepG2) and in a normal liver cell line (LO2) (Fig. S1D–F). The expression of *ANGPTL8* was significantly increased in the highly malignant HCC cell line MHCC97H compared to an HCC cell line with low metastatic spread, MHCC97 L, and the expression in both of these cell lines was also significantly higher compared to LO2 normal liver cells. The above findings indicate that the expression level of *ANGPTL8* is closely associated with malignancy in HCC.

### *ANGPTL8* KO alleviates carcinogen-induced hepatocarcinogenesis

**Fig. 1:**
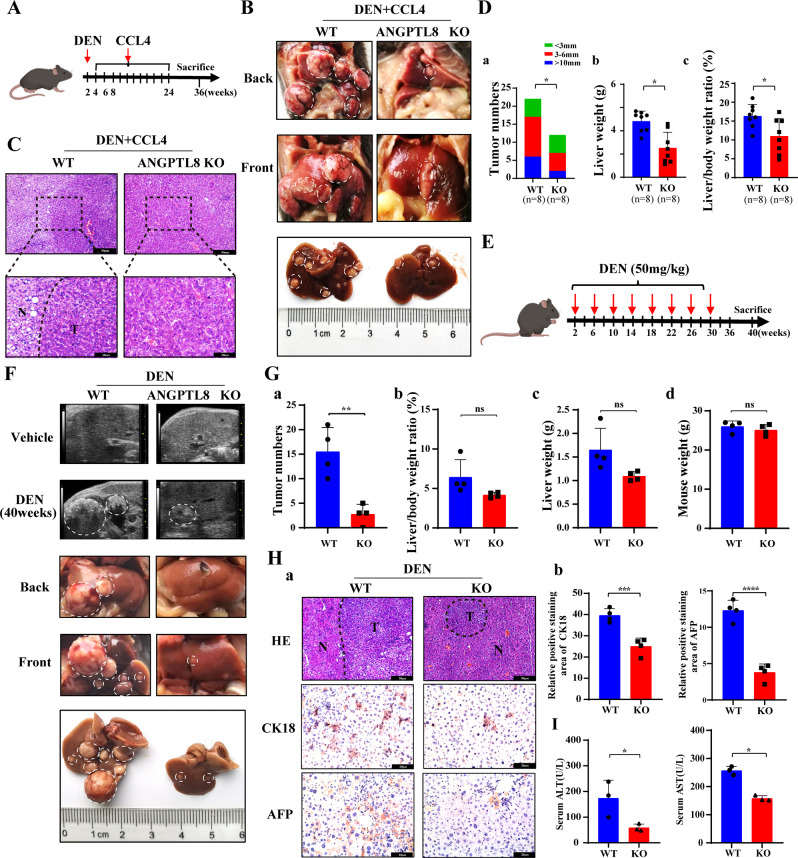
*ANGPTL8* KO alleviates the progression of DEN- and DEN-plus-CCL4-induced HCC. **A** Schematic illustration of the DEN-plus-CCL4-induced HCC model (*n* = 8 per group). **B** Gross appearance of livers from WT and *ANGPTL8*-KO mice induced with DEN plus CCL4. Tumors are indicated within white dashed lines (*n* = 8 per group). **C** Representative images of H&E-stained tissue samples isolated from DEN-plus-CCL4-induced HCC in WT and *ANGPTL8*-KO mice. T: tumor core area, N: adjacent nontumor area; scale bars, 50 and 20 μm (inset). **D** Statistical analysis of the differences in (a) tumor number, (b) liver weight, and (c) liver/body weight ratio between DEN-plus-CCL4-induced HCC WT and *ANGPTL8*-KO mice (*n* = 8 per group). Data are the mean ± SD. Statistical comparisons were performed using Student’s *t* test. *p < 0.05. **E** Schematic illustration of the DEN-induced HCC model (*n* = 12 per group). **F** Tumor formation was monitored by ultrasound, and the gross appearance of the livers was noted. Tumors are indicated within white dashed lines (*n* ≥ 4 per group). **G** Statistical analysis of differences in (a) tumor number, (b) liver/body ratio, (c) liver weight, and (d) mouse weight between DEN-induced WT and *ANGPTL8*-KO HCC mice (*n* = 4 per group). Data are the mean ± SD. Statistical comparisons were performed using Student’s *t* test. **p < 0.01). **H** (a) Representative images of H&E staining and IHC staining of CK18 and AFP in liver tissues from DEN-induced WT and *ANGPTL8*-KO HCC mice. T: tumor core area, N: adjacent nontumor area; scale bars, 50 and 20 μm (inset). (b) Statistical analysis of CK18- and AFP-positive hepatocytes between DEN-induced WT and *ANGPTL8-*KO HCC mice (*n* = 4 per group). Data are the mean ± SD. Statistical comparisons were performed using Student’s *t* test. ***p < 0.001, ****p < 0.0001. **I** Serum ALT and AST levels were measured 40 weeks post DEN treatment (*n* = 3 per group). Data are the mean ± SD. Statistical comparisons were performed using Student’s *t* test. *p < 0.05.

To understand how ANGPTL8 might affect early tumor development, we evaluated different models of hepatocarcinogenesis. Studies have indicated that hepatic fibrosis can progress to cirrhosis and eventually to HCC [21]; therefore, we first characterized a mouse model of liver fibrosis. CCL4 was injected into the liver of 6-week-old WT C57BL/6J mice or isogenic *ANGPTL8*-KO mice, and animals were euthanized after 8 weeks to examine the liver tissue. The livers from WT mice were more fibrotic than those from *ANGPTL8*-KO mice, and CCL4 upregulated the expression of ANGPTL8 in mouse livers, suggesting that ANGPTL8 promotes the progression of CCL4-induced liver fibrosis (Fig. S2A–D). We therefore speculated that ANGPTL8 may play a role in hepatocarcinogenesis, and results showed that DEN, a known chemical carcinogen that induces hepatic tumors similar to HCC, upregulated *ANGPTL8* expression in the livers of mice (Fig. S2E), so we next induced hepatocarcinogenesis by combining DEN plus CCL4 treatment (Fig. 1A). Animals were monitored daily and euthanized at 36 weeks of age. The livers were collected to note the gross appearance (Fig. 1B), and then H&E staining was performed to identify tumor tissue (Fig. 1C). *ANGPTL8*-KO mice had significantly fewer and smaller tumors than WT mice (Fig. 1D). Next, we repeated a similar experiment with DEN alone using a single 50 mg/kg injection into the liver of 2-week-old animals (Fig. 1E). Tumor development was monitored by ultrasound imaging, which revealed that the livers of WT mice had uneven edges, abnormal parenchymal morphology, and space-occupying lesions, whereas the livers of *ANGPTL8*-KO mice had a uniform texture, normal size and shape, no obvious ascites or peripheral lymphatic enlargement, and no obvious space-occupying lesions (Fig. 1F). Furthermore, the number of nodules present in WT mice was significantly greater than that in *ANGPTL8*-KO mice, but body weight, liver weight, and liver-to-body-weight ratio were similar between the two groups (Fig. 1G). Immunohistochemistry analysis indicated fewer CK18- and AFP-positive cells in liver slices from *ANGPTL8*-KO mice than in those from WT mice (Fig. 1H). Although the serum levels of AST and ALT were outside the normal range in both WT and KO mice, ANGPTL8 KO mice had lower levels of serum ALT and AST than that of WT mice (Fig. 1I). These results suggest that *ANGPTL8* KO inhibits the development of DEN-induced and DEN plus CCL4-induced HCC.

### ANGPTL8 promotes proliferation in normal liver cells and in HCC cells

**Fig. 2:**
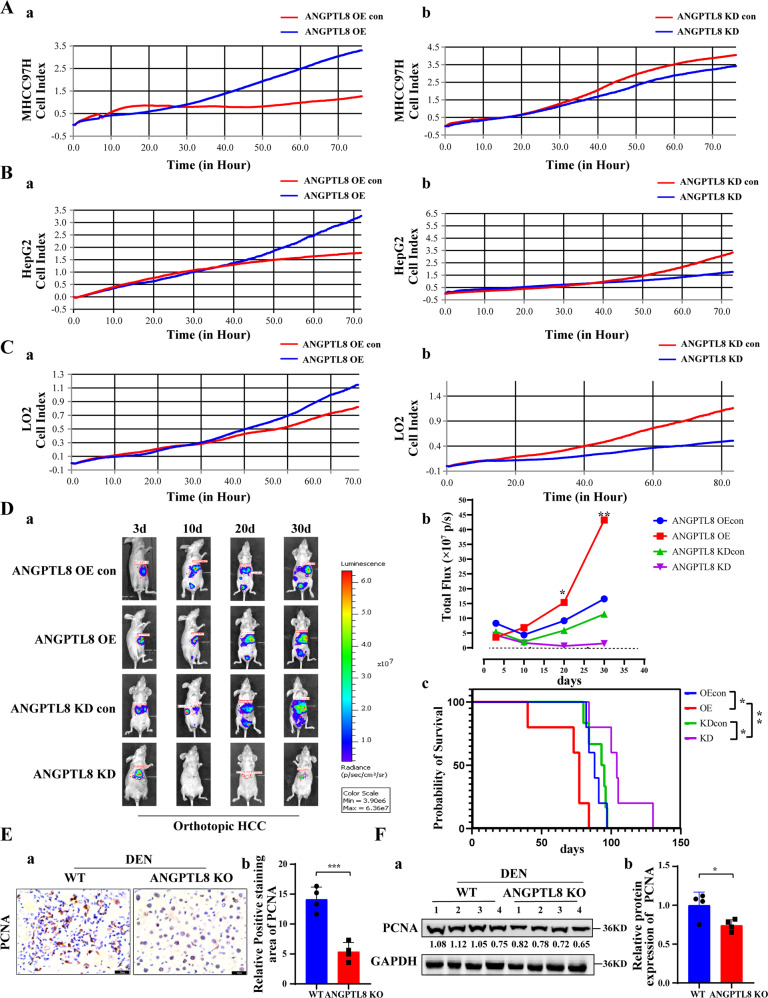
ANGPTL8 promotes proliferation in HCC and normal liver cells in vitro and in vivo. **A**–**C** Proliferation of **A** MHCC97H, **B** HepG2, and **C** LO2 cells with *ANGPTL8* OE or KD by RTCA. **D** (a, b) Analysis of the growth of orthotopically implanted WT, *ANGPTL8*-KD or *ANGPTL8-*OE MHCC97H-Luc tumor cells by bioluminescence imaging (*n* ≥ 5 per group). (c) For the survival experiments, the time of death of every mouse was recorded after the orthotopic tumor formation experiment in nude mice. Data are the mean ± SD. Statistical comparisons were performed using Student’s *t* test. *p < 0.05, **p < 0.01. **E** IHC staining of PCNA in liver tissue from DEN-induced WT and *ANGPTL8*-KO HCC mice (*n* = 4 per group). Data are the mean ± SD. Statistical comparisons were performed using Student’s *t* test. ***p < 0.001. **F** Western blot analysis of PCNA expression in liver tissue from DEN-induced WT and *ANGPTL8*-KO HCC mice (*n* = 4 per group). Protein expression was normalized to GAPDH, and the numbers represent the mean ± SD from an average of 4 independent experiments. Statistical comparisons were performed using Student’s *t* test. *p < 0.05.

Abnormal cell proliferation is one of the most important characteristics of cancer cells. To explore the effect of ANGPTL8 on the proliferation of HCC cells in vitro and on tumorigenic capacity in vivo, we engineered MHCC97H and HepG2 HCC cells as well as LO2 normal liver cells with lentivirus-mediated CRISPR-Cas9 or with an *ANGPTL8* OE vector. *ANGPTL8* OE or addition of human ANGPTL8 recombinant protein (rANGPTL8, 500 ng/mL) to the cell culture medium promoted proliferation in both HCC cell lines and LO2 cells (Figs. 2A–C and S3). To examine the effects in vivo, luciferase-labeled MHCC97H cells (MHCC97H-Luc) with *ANGPTL8* KD or OE constructs or with nontargeting/empty vectors were orthotopically injected into the livers of nude mice, and tumor development was monitored using bioluminescence imaging. We observed significantly higher luciferase activity in *ANGPTL8*-OE tumors than in control tumors, whereas *ANGPTL8* KD significantly decreased the luciferase signal compared to that in control tumors. The overall survival time of the ANGPTL8-OE group mice was significantly shorter than that of the control group mice, while that of the ANGPTL8 KD group mice was significantly longer (Fig. 2D). Moreover, immunohistochemistry and western blotting showed that *ANGPTL8* KD inhibited hepatocyte proliferation in the DEN-induced HCC mouse model (Fig. 2E, F). Taken together, these findings suggest that ANGPTL8 promotes proliferation in normal liver cells and in HCC cells in vitro and accelerates HCC growth in vivo.

### ANGPTL8 promotes ROS accumulation in early-stage DEN-induced HCC

Compensatory proliferation after liver damage has been suggested to be mediated by inflammatory cytokines [22, 23], which serve as a main driver of DEN-induced HCC [24]. In the liver, ANGPTL8 is extensively expressed in parenchymal and a few non-parenchymal cells, with higher expression in hepatocytes than Kupffer cells but rarely in stellate cells (Fig. S4). To study the effect of ANGPTL8 on these responses, we injected DEN into 15-day-old mice and detected liver function-related indicators at different time points. *ANGPTL8* KO significantly alleviated liver injury (Fig. S5A, B) and significantly reduced liver infiltration by inflammatory mediators (Fig. S5C) 48 h after DEN administration. *ANGPTL8* KO also reduced the release of the inflammatory cytokines IL-6 and IL-1β (Fig. S5D). The accumulation of reactive oxygen species (ROS) is an important factor in inflammation [25, 26]. Therefore, we examined ROS levels in primary liver cells from mice 48 hours after DEN administration using the DCFH-DA fluorescence assay. ROS accumulation was significantly lower in liver cells from *ANGPTL8*-KO mice than in those from WT mice, and culturing *ANGPTL8*-KO cells with rANGPTL8 protein rescued the phenotype and restored ROS to WT levels (Fig. S5E, F). Together, these data indicate that ANGPTL8 promotes the inflammatory response in perturbed liver tissue by regulating ROS levels, which contributes to the early stages of DEN-induced HCC.

### ANGPTL8 activates ROS/ERK pathway-mediated autophagy to promote HCC cell proliferation

**Fig. 3:**
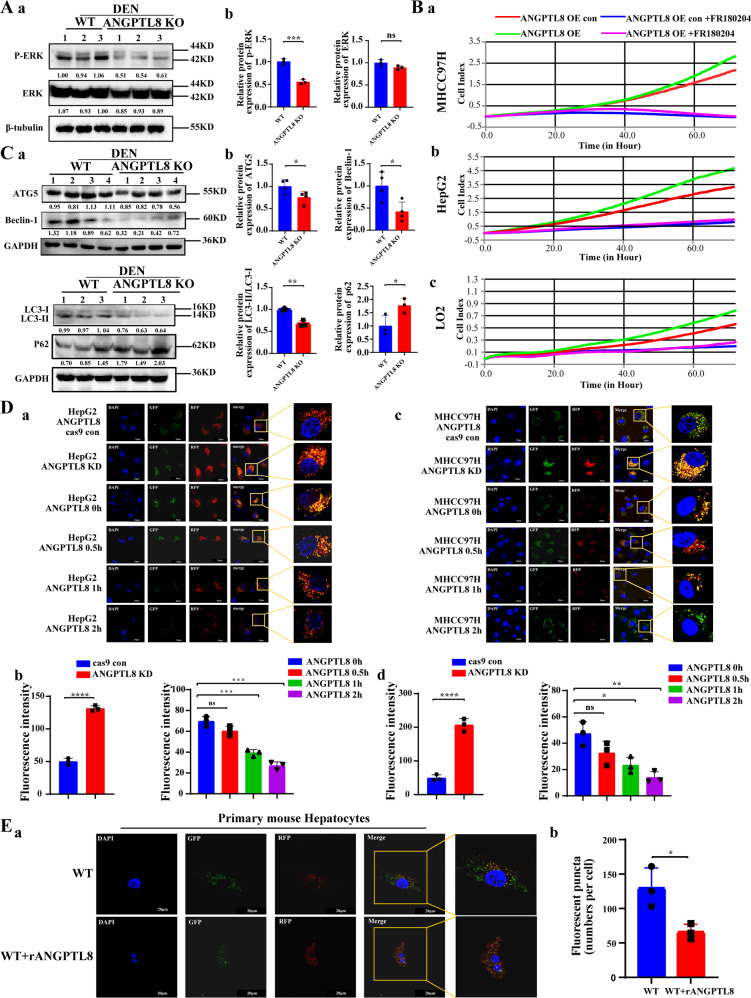
ANGPTL8 regulates autophagy through the ROS/ERK pathway in HCC cells. **A** Detection of phosphorylated and total ERK (P-ERK and ERK) in the liver tissue of DEN-induced WT and *ANGPTL8*-KO HCC mice by western blotting (*n* = 3 per group). Protein expression was normalized to β-tubulin, and the numbers represent the mean ± SD of an average of 3 independent experiments. ***p < 0.001. **B** RTCA monitoring of the proliferation of MHCC97H, HepG2, and LO2 cells with *ANGPTL8* OE or KD and with or without ERK inhibitor treatment (FR180204) for 24 h. **C** ATG5, Beclin-1, LC3II/I and P62 levels in the liver tissue of DEN-induced WT and *ANGPTL8*-KO HCC mice detected by western blotting (*n* ≥ 3 per group). Protein expression was normalized to GAPDH, and the numbers represent the mean ± SD from an average of 3 independent experiments. Statistical comparisons were performed using Student’s *t* test. *p < 0.05, **p < 0.01. **D**, **E** The autophagic flux in **D** (a, b) HepG2, **D** (c, d) MHCC97H, and **E** primary mouse hepatocytes in *ANGPTL8* knockout cells or in cells incubated with rANGPTL8 (500 ng/mL) was monitored using the fluorescence reporter Adplus-mCherry-GFP-LC3B. Scale bar, 20 μm. Autophagic flux was evaluated by calculating the number of red dots (autolysosomes) and yellow dots (autophagosomes) (*n* = 3 per group). Data are the mean ± SD. Statistical comparisons were performed using Student’s *t* test. *p < 0.05, **p < 0.01, ***p < 0.001, ****p < 0.0001.

To delineate which downstream signaling pathways are activated by ROS to promote HCC cell proliferation, we collected proteins from *ANGPTL8*-OE and control HepG2 cells and conducted protein array analysis to detect changes in the expression of total and phosphorylated proteins. *ANGPTL8* OE increased the phosphorylation of ERK1/2 (T202/Y204) compared to that in control cells but did not affect the total expression of these proteins (Fig. S6A). Studies have shown that ROS can regulate the ERK signaling pathway to promote the proliferation of HCC cells [27], and our previous study demonstrated that ANGPTL8 promotes liver fibrosis in NAFLD through the ERK pathway [13]. Therefore, we speculated that ANGPTL8 may induce abnormal proliferation of hepatocytes by regulating the ERK pathway. ERK phosphorylation was markedly lower in liver tissue collected from DEN-treated *ANGPTL8*-KO mice than in liver tissue from WT mice (Fig. 3A). Consistently, *ANGPTL8* OE or *ANGPTL8* KD increased or decreased ERK phosphorylation, respectively, in HCC cell lines and in primary liver cells, and adding rANGPTL8 to the medium similarly increased ERK phosphorylation (Fig. S6B–D). To determine whether our findings in *ANGPTL8*-OE models could be attributed to ERK signaling, we treated HCC and primary liver cells overexpressing *ANGPTL8* with the ERK inhibitor FR180204 and measured cell proliferation. Consistent with our hypothesis, FR180204 significantly decreased proliferation in HCC and primary liver cells compared to untreated cells (Fig. 3B).

**Fig. 4:**
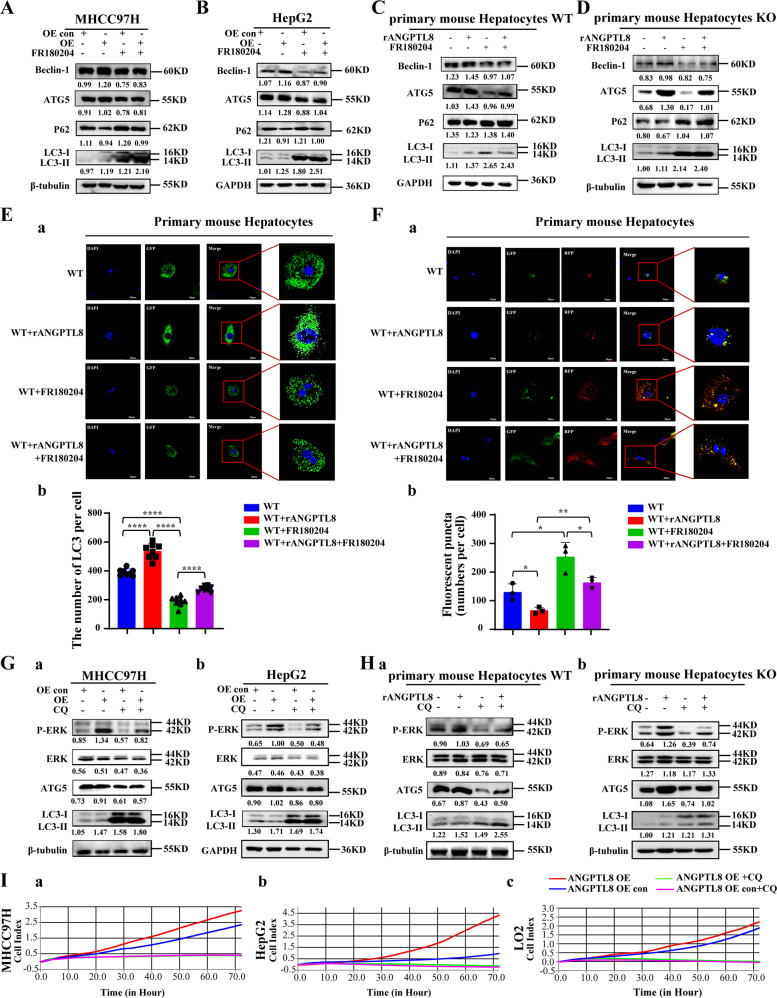
ANGPTL8 activates ERK pathway-mediated autophagy and promotes the proliferation of HCC cells. **A**, **B** Beclin-1, ATG5, P62, and LC3II/I levels in *ANGPTL8*-overexpressing **A** MHCC97H and **B** HepG2 cells treated with or without the ERK inhibitor FR180204 were detected by western blotting. Protein expression was normalized to β-tubulin or GAPDH, and the numbers represent the mean ± SD of the average of 3 independent experiments. **C**, **D** Beclin-1, ATG5, P62, and LC3II/I levels in **C** WT and **D** *ANGPTL8*-KO primary mouse hepatocytes cultured with or without rANGPTL8 and FR180204 were detected by western blotting. Protein expression was normalized to β-tubulin or GAPDH, and the numbers represent the mean ± SD of an average of 3 independent experiments. **E** (a) Autophagy levels in primary mouse hepatocytes treated with rANGPTL8 or the ERK inhibitor FR180204 were monitored using the fluorescence reporter Ad-GFP-LC3B, and (b) autophagy levels were quantified by calculating the number of green dots (*n* = 8 per group). Data are the mean ± SD. Statistical comparisons were performed using Student’s *t* test. ****p < 0.0001. **F** (a) Autophagic flux in primary mouse hepatocytes treated with rANGPTL8 or the ERK inhibitor FR180204 was monitored using the fluorescence reporter Adplus-mCherry-GFP-LC3B. (b) Autophagy levels were quantified by calculating the number of red and yellow puncta (*n* = 3 per group). Data are the mean ± SD. Statistical comparisons were performed using Student’s *t* test. *p < 0.05, **p < 0.01. **G** Phosphorylated ERK (P-ERK), ERK, ATG5 and LC3II/I in *ANGPTL8*-OE (a) MHCC97H and (b) HepG2 cells with or without the autophagy inhibitor CQ (50 μΜ, 24 h) were detected by western blotting. **H** Phosphorylated ERK (P-ERK), ERK, ATG5 and LC3II/I in primary mouse hepatocytes from (a) WT and (b) *ANGPTL8*-KO mice with or without rANGPTL8 and CQ were detected by western blotting. **I** The proliferation of *ANGPTL8*-overexpressing MHCC97H, HepG2, and LO2 cells treated with or without CQ was monitored by RTCA.

ERK phosphorylation can activate autophagy to promote the proliferation of HCC cells and HCC progression; [28] therefore, we characterized the expression of autophagy-related proteins in liver tissue from DEN-induced mice. ATG5, Beclin-1, and LC3 II/I levels were significantly lower and P62 expression was significantly higher in livers from *ANGPTL8*-KO mice than in livers from WT mice (Fig. 3C). Furthermore, *ANGPTL8* OE or the addition of rANGPTL8 to the culture of HCC and primary liver cells significantly upregulated the expression of autophagy-related proteins (Fig. S7). Next, we evaluated the effect of ANGPTL8 on autophagic flux by infecting our engineered HCC and primary liver cell lines with the Adplus-mCherry-GFP-LC3B vector, a dual fluorescence reporter, to detect autophagosomes. *ANGPTL8* OE or addition of rANGPTL8 to the culture medium both increased the number of autophagosomes compared to the parental cell lines or untreated cells, whereas the number of autophagosomes was decreased in *ANGPTL8*-KD cells compared with parental cells (Fig. 3D, E), supporting a role for ANGPTL8 in regulating autophagic flux. To determine whether ANGPTL8 modulates autophagy through the ERK pathway, we repeated these experiments in cultures with or without the ERK inhibitor FR180204. In HCC and primary liver cells overexpressing *ANGPTL8*, the presence of FR180204 increased the amount of LC3II/I and decreased the amount of ATG5 and Beclin-1 (Figs. 4A–D and S8), and FR180204 reduced the number of autophagosomes induced by rANGPTL8 (Figs. S9 and 4E, F). These results suggest that ANGPTL8 regulates autophagy through the ERK signaling pathway.

To determine the stage of autophagy at which ANGPTL8 participates, HCC cells were cultured with rANGPTL8 in addition to 3-methyladenine (3MA), an early-stage autophagy inhibitor, or with chloroquine (CQ), a late-stage autophagy inhibitor. While 3MA did not affect ANGPTL8-mediated autophagy activation (Fig. S10), CQ significantly attenuated the effect of ANGPTL8 on autophagy (Fig. 4G, H and S11). Moreover, CQ eliminated the hyperproliferative phenotype induced by ANGPTL8 in HCC cells (Fig. 4I), which strongly suggests that ANGPTL8 promotes cell proliferation by activating autophagy and by modulating the ERK signaling pathway.

### ANGPTL8 mediates the immune escape of HCC cells by upregulating *Fgr* expression and inducing macrophage polarization

**Fig. 5:**
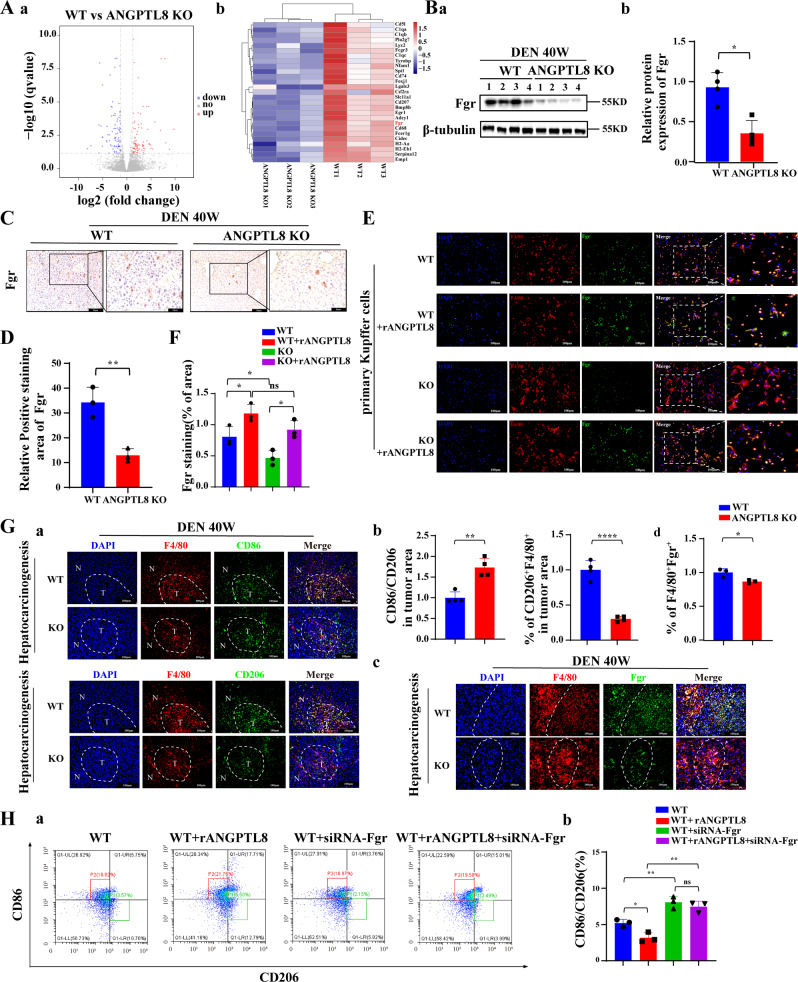
ANGPTL8 upregulates *Fgr* expression and promotes macrophage polarization to the M2 phenotype. **A** (a) Volcano plot of all differentially expressed genes in RNA-seq analysis of liver tissue from DEN-induced WT and *ANGPTL8*-KO HCC mice (*n* = 3 per group). (b) Heatmap of all differentially expressed genes in RNA-seq analysis of livers from DEN-induced WT and *ANGPTL8*-KO HCC mice (*n* = 3 per group). **B** Fgr expression in the livers of DEN-induced WT or *ANGPTL8*-KO HCC mice detected by western blotting (*n* = 4 per group). Protein expression was normalized to β-tubulin, and the numbers represent the mean ± SD from an average of 3 independent experiments. Statistical comparisons were performed using Student’s *t* test. *p < 0.05. **C**, **D** Representative images of IHC staining of Fgr in the livers of DEN-induced WT and *ANGPTL8*-KO HCC mice (*n* = 3 per group). Data are the mean ± SD. Scale bars, 20 and 50 μm (inset). Statistical comparisons were performed using Student’s *t* test. **p < 0.01. **E**, **F** Representative images of immunofluorescence staining of *Fgr* in primary Kupffer cells from WT and *ANGPTL8*-KO mice (*n* = 3 per group). Scale bar, 100 μm. Data are the mean ± SD. Statistical comparisons were performed using Student’s *t* test. *p < 0.05. **G** (a, b) Representative images of immunofluorescence staining of CD86, CD206, and F4/80 in the liver tissue of DEN-induced WT and *ANGPTL8*-KO HCC mice. (c, d) Representative images of immunofluorescence staining of Fgr in the liver tissue of DEN-induced WT and *ANGPTL8*-KO HCC mice. In all images, the dotted white line represents the edge of the tumor. T: tumor core area, N: adjacent nontumor area. Scale bar, 100 μm. Data represent the mean ± SD from experiments with *n* ≥ 3 per group. Statistical comparisons were performed using Student’s *t* test. *p < 0.05, **p < 0.01, ****p < 0.0001. **H** Flow cytometry analysis of CD86 and CD206 in RAW264.7 cells with or without rANGPTL8 and *Fgr* siRNA (*n* = 3 per group). Data are the mean ± SD. Statistical comparisons were performed using Student’s *t* test. *p < 0.05, **p < 0.01.

Abnormal cell proliferation and escape from immune surveillance are the fundamental causes of tumor occurrence. To identify genes that are regulated by ANGPTL8 and may affect cell proliferation or immune clearance of HCC cells, we performed RNA sequencing (RNA-seq) analysis on liver tumors collected from *ANGPTL8*-KO and WT mice 40 weeks after treatment with DEN. We found that the differentially expressed genes in *ANGPTL8*-KO tumors were mainly involved in the immune response (Fig. 5A), and we speculated that ANGPTL8 may modulate the immune system to facilitate the escape of abnormally proliferating cells from immune surveillance. Then, we validated the expression levels of dysregulated genes by RT‒qPCR, which showed that *Fgr*, a member of the Src family of nonreceptor tyrosine kinases, was the most significantly downregulated gene in *ANGPTL8*-KO tumors (Fig. S12A, B). Western and immunohistochemistry analyses also confirmed decreased *Fgr* protein levels in *ANGPTL8*-KO tumors compared to WT tumors (Fig. 5B–D). Next, we analyzed the tumor tissue using a single-cell sequencing library, which showed that *Fgr* was expressed mainly in immune cells (Figure S12C). Additionally, *Fgr* was highly expressed in Kupffer cells, which are resident infiltrating macrophages in the liver, but its expression was not detected in primary liver cells (Fig. S12D). Consistent with these results, the addition of rANGPTL8 to primary Kupffer cells in vitro also upregulated *Fgr* expression (Fig. 5E, F). Further clinical studies revealed that the expression of ANGPTL8 and Fgr was positively correlated in HCC tissues (Fig. S13).

To more specifically determine the effect of ANGPTL8 on the immune compartment in HCC, we used immunofluorescence to characterize immune cells in *ANGPTL8*-KO and WT tumors. In liver tissue collected from DEN-induced HCC mice at 10 months of age, the expression of CD86, an M1-type cell marker, and the ratio of CD86/CD206 were increased in *ANGPTL8*-KO tissue compared to WT tissue. Furthermore, the expression of CD206, an M2-type cell marker, F4/80, and *Fgr* was lower in *ANGPTL8*-KO tissue than in WT tissue (Fig. 5G). To study the effect of *Fgr* on the M1/M2 polarization of macrophages, we inhibited the expression of *Fgr* using *Fgr*-targeted siRNA or the *Fgr* inhibitor AZD0530 in RAW264.7 macrophages and primary Kupffer cells incubated with rANGPTL8. Both genetic and pharmacological inhibition of *Fgr* prevented ANGPTL8-induced macrophage polarization to the M2 phenotype (Figs. S14, S15, and 5H). Immunofluorescence analysis of DEN-induced mouse HCC tumors using T-cell markers also determined that the number of infiltrating immunosuppressive CD4^+^FOXP3^+^ and CD8^+^PD-1^+^ T cells was significantly reduced in *ANGPTL8*-KO tumors compared to WT tumors (Fig. S16). Taken together, these results suggest a model in which ANGPTL8 upregulates *Fgr* expression, which promotes macrophage polarization to the M2 phenotype and concomitantly regulates tumor-associated T-cell function to create an immunosuppressive microenvironment that allows pathogenic HCC cells to escape immune surveillance.

### ANGPTL8 interaction with LILRB2/PIRB is required for its protumorigenic effects

**Fig. 6:**
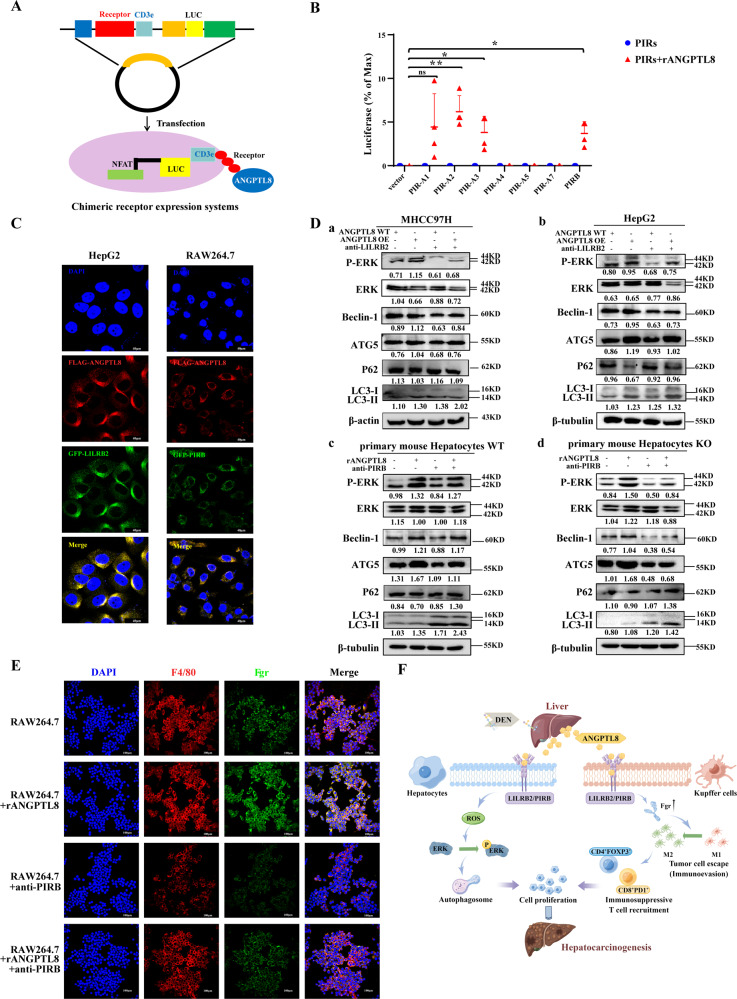
The ANGPTL8 interaction with the LILRB2/PIRB receptor regulates the ERK pathway in HCC cells and *Fgr* expression in Kupffer cells. **A** Schematic diagram of the PIR receptor interaction with ANGPTL8 based on the chimeric luciferase receptor system. **B** The interaction between ANGPTL8 and PIRs was detected using a chimeric luciferase receptor assay (*n* = 4 per group). Data are the mean ± SD. Statistical comparisons were performed using Student’s *t* test. *p < 0.05, **p < 0.01. **C** ANGPTL8 and LILRB2/PIRB expression in HepG2 and RAW264.7 cells detected by immunofluorescence using ANGPTL8-Flag and LILRB2/PIRB-GFP plasmids. Scale bar, 40 μm. **D** P-ERK, ERK, LC3II/I, ATG5, P62 and Beclin-1 expression detected by western blotting in (a) MHCC97H, (b) HepG2, (c) WT, and (d) *ANGPTL8*-KO (d) primary mouse hepatocytes with *ANGPTL8* OE or rANGPTL8 during blocking using an anti-LILRB2/PIRB antibody. Protein expression was normalized to β-actin or β-tubulin, and the numbers represent the mean ± SD of an average of 3 independent experiments. **E** Representative images of cellular immunofluorescence staining of *Fgr* in RAW264.7 cells with or without rANGPTL8 and PIRB antibody treatment. Scale bar, 100 μm. **F** Schematic representation of the mechanism by which ANGPTL8 accelerates hepatocarcinogenesis by promoting tumor cell proliferation and modulating macrophage polarization to facilitate immune escape.

The PIRs in rodents or leukocyte Ig-like receptors (LILRs) in humans are specific receptors of ANGPTLs [29]. Therefore, we used a luciferase-based chimeric receptor assay to analyze potential interactions between ANGPTL8 and PIRs in 293T cells. ANGPTL8 interacted with PIRA2, PIRA3, and PIRB but not with PIRA4, PIRA5, or PIRA7 (Fig. 6A, B). Previous studies have demonstrated that human or rat LILRB2 and its mouse ortholog, PIRB, are receptors for ANGPTLs. Consistently, immunofluorescence analysis of HCC cells and RAW264.7 macrophages showed that ANGPTL8 colocalized with LILRB2 on the cell membrane (Fig. 6C). Moreover, blocking LILRB2 with an antibody inhibited ANGPTL8-induced ROS accumulation in ANGPTL8 OE MHCC97H cells (Fig. S17). Then, in HCC and primary liver cells with *ANGPTL8* OE or cultured with rANGPTL8, treatment with an anti-LILRB2/PIRB antibody inhibited ERK phosphorylation and autophagy (Figs. 6D and S18). Finally, we demonstrated that the ANGPTL8-induced upregulation of *Fgr* expression in RAW264.7 macrophages was also inhibited by treatment with an anti-LILRB2/PIRB antibody (Fig. 6E). Together, these ANGPTL8-mediated activities create a permissive microenvironment for tumorigenic hepatocytes to evade immune surveillance and drive HCC initiation and progression.

## Discussion

Despite a significant decline in the mortality rate of many types of cancers over the past decade, the mortality rate for patients with HCC has not meaningfully improved, and HCC is still the third-highest cause of cancer-related death [30]. Accumulating evidence suggests that hepatic inflammation induced upon liver injury drives HCC development by promoting the survival and proliferation of hepatocytes harboring oncogenic mutations. However, less is known regarding the role of hepatic inflammation in mediating crosstalk between HCC cells and other cells in the tumor microenvironment. In this study, we identified ANGPTL8 as a key signaling molecule in HCC cells and immune cells-particularly macrophages-during hepatocarcinogenesis. We demonstrate that ANGPTL8 interacts with the LILRB2/PIRB receptor to execute crucial activities that promote tumor cell proliferation and modulate macrophage polarization and T-cell infiltration to facilitate the immune escape of tumorigenic cells (Fig. 6F), making ANGPTL8 an attractive therapeutic target for treating HCC.

ANGPTL8 has proinflammatory functions, and our previous study showed that ANGPTL8 was upregulated in the serum of patients with hepatic fibrosis and was higher than that in patients with HCC [13]; however, a previous study did not explore the role of ANGPTL8 in hepatocarcinogenesis. In this study, we found that the level of increase in ANGPTL8 expression was positively correlated with the degree of HCC malignancy, and high ANGPTL8 was associated with poor prognosis. The most important characteristics of malignant tumors are uncontrolled cell proliferation and infinite tumor growth [31]. Abnormal expression of tumor genes leads to aberrant cell proliferation that can stimulate tumor development; [32] thus, we explored the effect of ANGPTL8 on HCC cell proliferation in cell line models of HCC and primary liver cells engineered with genetic knockdown or overexpression constructs and exposed to exogenous rANGPTL8. These studies showed that endogenous and exogenous ANGPTL8 promoted HCC cell proliferation in vitro and in vivo.

To explore the role of ANGPTL8 in hepatocarcinogenesis, we established DEN/CCL4-induced and DEN-induced murine models of HCC and found that *ANGPTL8* KO inhibited HCC development in both models. These results demonstrate that ANGPTL8 plays an important role in HCC development, but the underlying mechanism by which ANGPTL8 exerts oncogenic functions in HCC remains unclear. Studies have shown that DEN stimulates an inflammatory response and hyperproliferation that help drive tumor progression by increasing ROS generation [24]. We found that DEN could robustly upregulate inflammatory cytokines and the expression of ANGPTL8 and that ANGPTL8 could increase ROS levels in PMHs. Studies have shown that ROS induce ERK phosphorylation to promote abnormal hepatocyte proliferation and hepatocarcinogenesis [33]. In our study of DEN-induced HCC in mice, we found that *ANGPTL8* KO reduced the levels of ROS and phosphorylated ERK. In vitro, treating HCC and liver cells with an ERK inhibitor attenuated *ANGPTL8* OE-induced cell proliferation.

ERK phosphorylation modulates autophagy [34, 35], and autophagy has been shown to be associated with cell proliferation in cancer cells [36]. Therefore, we speculated that ANGPTL8-mediated activation of the ERK pathway could promote HCC cell proliferation via autophagy. Indeed, *ANGPTL8* OE increased autophagic flux, and this effect was reversed by adding an ERK inhibitor. The proliferative capacity of HCC cells overexpressing *ANGPTL8* was also inhibited by adding an inhibitor of autophagic flux, indicating that DEN-induced upregulation of *ANGPTL8* expression activates the ROS/ERK pathway, which induces autophagy and promotes HCC cell proliferation and hepatocarcinogenesis.

Normally, a healthy immune system can clear abnormally proliferating cells, thereby impeding the formation of tumors [27, 37]. The ability to escape immune surveillance is critical for tumor development [38], and our RNA-seq results showed that ANGPTL8 modulates genes involved in the immune response in DEN-induced mouse liver tumors. Among the dysregulated genes, *ANGPTL8* KO most significantly downregulated the expression of the oncogene *Fgr*. Many studies have shown that *Fgr* promotes tumor progression and metastasis by inducing macrophage polarization into the M2 phenotype to promote tumor cell escape from immune surveillance [39]. Our RNA-seq results also showed that ANGPTL8 can significantly upregulate CD68 expression, suggesting that ANGPTL8 may recruit a large number of macrophages into liver cancer tissues, so we next compared the number of Kupffer cells, which are the resident macrophage cells of the liver, between DEN-induced tumors from WT and *ANGPTL8*-KO mice. In agreement with the results from RNA-seq analysis, the number of F4/80-positive cells was significantly reduced in the liver tumors of *ANGPTL8*-KO mice compared to WT mice. Furthermore, we demonstrated that ANGPTL8 inhibited macrophage polarization into the M1 phenotype and coordinately regulated tumor-associated T cell function to form an immunosuppressive microenvironment, leading to the escape of tumorigenic HCC cells from immune surveillance. These results suggest that ANGPTL8 is an important regulator of the tumor immune microenvironment and promotes hepatocarcinogenesis.

Our previous study identified a direct interaction between ANGPTL8 and the PIRB receptor [16], which corresponds to LILRB2 in humans [40]. LILRB2/PIRB is an inhibitor of inflammation and autoimmunity [41], and LILRB2 blockade with antagonizing antibodies increases tumor cell death and the killing activity of cytotoxic T lymphocytes [20]. We found that ANGPTL8 colocalized with LILRB2/PIRB on the cell membrane, and a chimeric receptor assay confirmed a direct interaction between them. We propose that the ANGPTL8-LILRB2/PIRB interaction may have a dual role in hepatocarcinogenesis. First, the high expression of ANGPTL8 could increase interactions with PIRB in hepatocytes to activate the ERK signaling pathway, leading to autophagy and ultimately promoting HCC cell proliferation. Second, ANGPTL8 could also interact with PIRB in macrophages (Kupffer cells) to upregulate *Fgr* expression and drive macrophage polarization into the M2 phenotype to suppress anti-tumor immune responses by increasing the number of CD4^+^FOXP3^+^ and CD8^+^PD-1^+^ T cells in the tumor microenvironment. Kupffer cells are critical immune cells in the HCC microenvironment, and they facilitate HCC growth [42]. HCC tumor-associated Kupffer cells include two major subpopulations: tumor-supportive M2 macrophages and tumor-suppressive M1 macrophages [43]. M2 macrophages are immunosuppressive [44], which facilitates the escape of abnormally proliferating hepatocytes from immune surveillance and eventually accelerates HCC development [45]. Based on our results, targeting the ANGPTL8–LILRB2/PIRB signaling axis may represent an attractive therapeutic strategy for HCC.

In the present study, we demonstrated the significance and mechanism of ANGPTL8 in promoting HCC pathogenesis. Notably, the mice used to establish HCC models were conventional *ANGPTL8*-KO mice, but were not hepatocyte- or liver-specific *ANGPTL8*-KO mice. Studies have revealed that liver- and adipose-derived ANGPTL8 play distinct roles in regulating lipid metabolism and obesity [46]. Therefore, models in hepatocytes or liver-specific conditional *ANGPTL8*-KO mice should be pursued to study the effect of tissue-specific secretion of ANGPTL8 on promoting HCC. Additionally, we showed that ANGPTL8 accelerated HCC progression through immune modulation, but we only investigated its role in the regulation of macrophage (Kupffer cell) polarization and the number of CD4^+^FOXP3^+^ and CD8^+^PD-1^+^ T cells. The functional effects of ANGPTL8 on other immune cells, such as myeloid-derived macrophages, dendritic cells, and natural killer cells, need to be further studied. Finally, as a highly expressed secretory protein in HCC cells, mechanistic studies to determine how ANGPTL8 precisely regulates interactions between HCC cells and other cells in the tumor immune microenvironment and whether it coincides with the site of LILRB2/PIRB binding when ANGPTL8 interacts with hepatocytes and Kupffer cells are important questions to be answered in future studies.

In summary, we demonstrate that the interaction of ANGPTL8 with LILRB2/PIRB induces hepatocyte and macrophage phenotypes that accelerate hepatocarcinogenesis. In hepatocytes, ANGPTL8-mediated stimulation of LILRB2/PIRB regulates the ROS/ERK pathway and upregulates autophagy, leading to HCC cell proliferation. In macrophages, ANGPTL8 binding to LILRB2/PIRB stimulates macrophage polarization to the M2 phenotype to suppress the antitumor immune response within the tumor microenvironment, allowing tumorigenic HCC cells to escape immune surveillance. We therefore propose *ANGPTL8* as a predictive marker for HCC development and a therapeutic target to treat HCC.

## Supplementary information

Supplementary materials & methods

## Data Availability

All of the data generated or analyzed in this study are included in this published article.
