# Supplementary material for: Dual role of ANGPTL8 in promoting tumor cell proliferation and immune escape during hepatocarcinogenesis
Source: Oncogenesis. 2023 May 15;12(1):26. doi: 10.1038/s41389-023-00473-3 (PMC10185523; doi:10.1038/s41389-023-00473-3)
Supplement: Supplementary file 1 — Supplementary materials & methods [file 41389_2023_473_MOESM1_ESM.docx]

**Supplementary materials & methods**

**Cell proliferation assay**

Cell proliferation was detected by real-time assessment using the xCELLigence real- time cell analyzer (RTCA, Roche, Sweden) and Cell Counting Kit-8 (CCK-8, New Cell & Molecular Biotech Co., Ltd., China) as previously described. HepG2, MHCC97H or LO2 cells (3×10^3^ cells) were added to the wells to make a final volume of 200 μL. All cells were allowed to settle at the bottom of the wells at RT for 15 min and then incubated at 37°C and 5% CO2. The impedance signals were recorded every 15 min for 72 h.

**Immunohistochemistry**

Immunohistochemistry analysis was performed as we previously reported. Tissues were embedded in paraffin and cut into 5-μm sections for hematoxylin and eosin (H&E) staining and immunohistochemistry (IHC). For IHC staining, the sections were incubated with primary antibodies against ANGPTL8 (ab180915, Abcam), PCNA (AF0239, Affinity), CK18 (10830-1-AP, Proteintech), AFP (abs135527, Absin), Fgr (abs115957, Absin), F4/80 (DF2789, Affinity) and IL-1β (12242S, CST) at 4°C overnight and then with HRP-conjugated secondary antibodies for 1 h followed by the Liquid DAB Substrate Chromogen System according to the manufacturer's instructions. The sections were examined under a fluorescence microscope (Olympus, Japan).

**Cellular immunofluorescence**

Cells were washed three times with PBS and fixed with an immunofluorescence fixator (Beyotime, P0098) at room temperature for 30 min. Fixed cells were permeabilized for 10 min. Cells were then blocked in 10% goat serum for 1 h at room temperature. Cells were stained with primary antibodies overnight at 4°C and then washed three times with PBS. Secondary antibodies were stained for 30 min at room temperature and then washed three times with PBS before DAPI Staining Solution (Beyotime, C1006) for 5 min. Cells were finally examined under a fluorescence confocal microscope (Olympus FV3000RS, Japan).

1

**Immunofluorescence for tissue sections**

For histological analysis, the large lobes of livers were dissected and fixed in a mix of 4% PFA and 30% sucrose in PBS overnight and then embedded in Tissue-Tek O.C.T. embedding compound. Blocks were frozen in a dry ice/ethanol bath. Sections (4.0 μm) were subjected to histological and immunochemical staining. Subsequent steps were performed as described above for cellular immunofluorescence. The primary antibodies were mouse anti-Albumin (66051-1-Ig, Proteintech), rat anti-F4/80 (ab6640, Abcam), rabbit anti-Fgr (abs115957, Absin), rabbit anti-CD86 (13395-1-AP, Proteintech), rabbit anti-CD206 (18704-1-AP, Proteintech), rat anti-FOXP3 (14-4776-82, ThermoFisher), rabbit anti-CD4 (ab183685, Abcam), mouse anti-CD8 (sc-7970, Santa Cruz), rabbit anti-PD1 (ab214421, Abcam), and rabbit anti-ANGPTL8 (ab180915, Abcam). Secondary antibodies were conjugated to Alexa Fluor 488 and Cy3 (A0423 and A0507, Beyotime). Fluorescence images were obtained using an Olympus Micro microscope (FV3000RS) and analyzed using Olympus Micro software (FV31S-SW).

**Flow cytometry**

The cells were preincubated with TruStain FcX™ PLUS (anti-mouse CD16/32) (BioLegend, 156603) antibody at 4°C for 10 min to block nonspecific binding and then stained with the following fluorochrome-conjugated antibodies at 4°C for 30 min: PE anti-mouse CD45 antibody (BioLegend, 103105), APC/Cyanine7 anti-mouse/human CD11b antibody (BioLegend, 101225), FITC anti-mouse F4/80 antibody (BioLegend, 123107), APC anti-mouse CD86 antibody (BioLegend, 105011), and PerCP/Cyanine5.5 anti-mouse CD206 (MMR) antibody (BioLegend, 141715), all purchased from BioLegend. The cells were detected on a CytoFlex Flow Cytometer (U.S. Beckman Coulter), and the data were processed using FlowJo software (Tree Star).

**Establishment of HCC cell lines with ANGPTL8 KD**

The sgRNA-coding cDNAs for targeting the ANGPTL8 gene were designed and synthesized to make the ANGPTL8-CRISPR-Cas9 constructs. The primers including the 20 bp target sequence and BsmBI sticky end were annealed and inserted into the lenti-CRISPRv2 plasmid (Genloci Biotechnologies, China) and digested with BsmBI

2

(NEB, USA). ANGPTL8-lenti-CRISPRv2/Cas9 plasmid was used for gene knockdown experiments. To package lentivirus, each lenti-CRISPRv2 plasmid with other components (psPAX2, pMD2.G) were transfected into HEK293T cells using Lipofectamine™ 3000 (Invitrogen, USA). Transfected cells were cultured in DMEM containing 5% FBS, 100 U/ml penicillin and 100 μg/ml streptomycin. Culture media were replaced after 24 h with fresh growth media. Forty-eight hours later, lentiviral particles were concentrated from culture media filtrated with 0.45 μm filters by using the Lenti-X Concentrator (Clontech, USA). Aliquots were stored at -80 °C until use. To transduce HepG2 and MHCC97H cells, 1.0 × 105 cells were plated in each well of a six-well plate, infected with the lentivirus, treated with polybrene for 24 hrsand selected by adding 3.5μg/ml puromycin to the growth medium for 4–6 days. Lentivirus with a scrambled sequence was used as control.The ANGPTL8 konckdown level was confirmed by western blotting.

**Establishment of HCC cell lines with ANGPTL8 OE**

HepG2 and MHCC97H cells were cultured until 70–80% confluency and then transcended with the lentiviruses ANGPTL8-GFP as per the manufacturer’s instructions. After 24 h of incubation, the medium was replaced with fresh medium containing 3.5μg/ml puromycin to initiate the positive HepG2 or MHCC97H cell screening procedure. On day 3-7, the cells transcended with lentiviruses ANGPTL8-
GFP (ANGPTL8OE) showed good growth and were observed under a microscope to confirm the presence of green fluorescence. ANGPTL8OE cells, identified by western blot assays, were selected for further experiments. The ANGPTL8 expression level was confirmed by western blotting.

**RNA-Seq data analysis**

RNA-seq of three pairs of WT and ANGPTL8 KO mouse primary liver cancer tissues was performed by Novogene using Illumina X TEN. The 6GB clean data per sample were collected for RNA-seq. Hg38 assembly was used for the read alignment, and gene annotation was obtained using Ensembl gene annotation version 90. Differential expression analysis was performed to yield statistically significant features (p-value < 0.05) between treatment groups using the Ballgown R package. The functional

3

annotation analysis was carried out using Ingenuity Pathway AnalysisTM and CIBERSORT analysis.

**ROS analysis**

The ROS levels were detected by an ROS assay kit (Beyotime, S0033S). Hepatocytes isolated from eight-week-old WT and *ANGPTL8* KO mice induced by DEN for 48 h were stained with 10 μM DCFH-DA for 30 min at 37°C in the dark. Then, the cell morphology was imaged by a fluorescence confocal microscope (Olympus FV3000RS, Japan), and the ROS level was quantified by flow cytometry (U.S. Beckman Coulter). **Measurement of autophagic flux**

The cells were transduced with Ad-GFP-LC3B (Beyotime, C3006) or Adplus- mCherry-GFP-LC3B (Beyotime, C3012) for 48 h followed by exposure to different conditions in different experiments. The cells were imaged by fluorescence microscopy, and the fluorescence intensity was measured from selected sections of five images per group using ZEN Pro software.

**siRNA knockdown**

The siRNAs targeting mouse *Fgr* were designed and synthesized by RiboBio Technology (Guangzhou, China).

Control siRNA, 5ʹ-TTCTCCGAACGTGTCACGTdTdT-3ʹ;

*Fgr* siRNA-1, 5ʹ-CTACATGGAAGTGAATGAT-3ʹ;

*Fgr* siRNA-2, 5ʹ-GAAGACTATTTCACCTCCA-3ʹ;

*Fgr* siRNA-3, 5ʹ-CCTAAAGGATCGAGAAGGT-3ʹ.

Transfection was carried out with Lipo8000™ (Beyotime, C0533). Forty-eight hours after transfection in RAW264.7 cells, western blotting assays were performed.
**Isolation of primary mouse liver cells**

Primary mouse hepatocytes (PMHs) and primary Kupffer cells (PKCs) were isolated by in situ retrograde perfusion of the liver with collagenase digestion medium, and the cell suspension was purified by discontinuous Percoll (Lablead, 17-0891-01) density gradient centrifugation as previously reported. Eight-week-old male C57BL/6J WT and ANGPTL8 KO mice were anesthetized with 1% sodium pentobarbital solution, and the hepatic portal vein was exposed with a ventral incision. A 24G catheter was inserted

4

into the portal vein and then secured with a vascular clamp. The liver was perfused with 30 mL of perfusion buffer HBSS and 0.5 mM EDTA, pH 7.4, at 2 mL/min, followed by 50 mL of digestion buffer DMEM/low glucose, 0.05% collagenase IV (Lablead, 17104-019), and 15 mM HEPES, pH 7.4, at 37 °C. After digestion, the cell suspension was filtered through 70 μm cell strainers and then centrifuged at 50 g for 2 minutes at 4°C to collect the PMH. The suspension was then subjected to density gradient centrifugation using Percoll to collect KCs, and the purity of the KCs was assessed by F4/80 staining. The viability of the cells was evaluated by trypan blue exclusion.

5


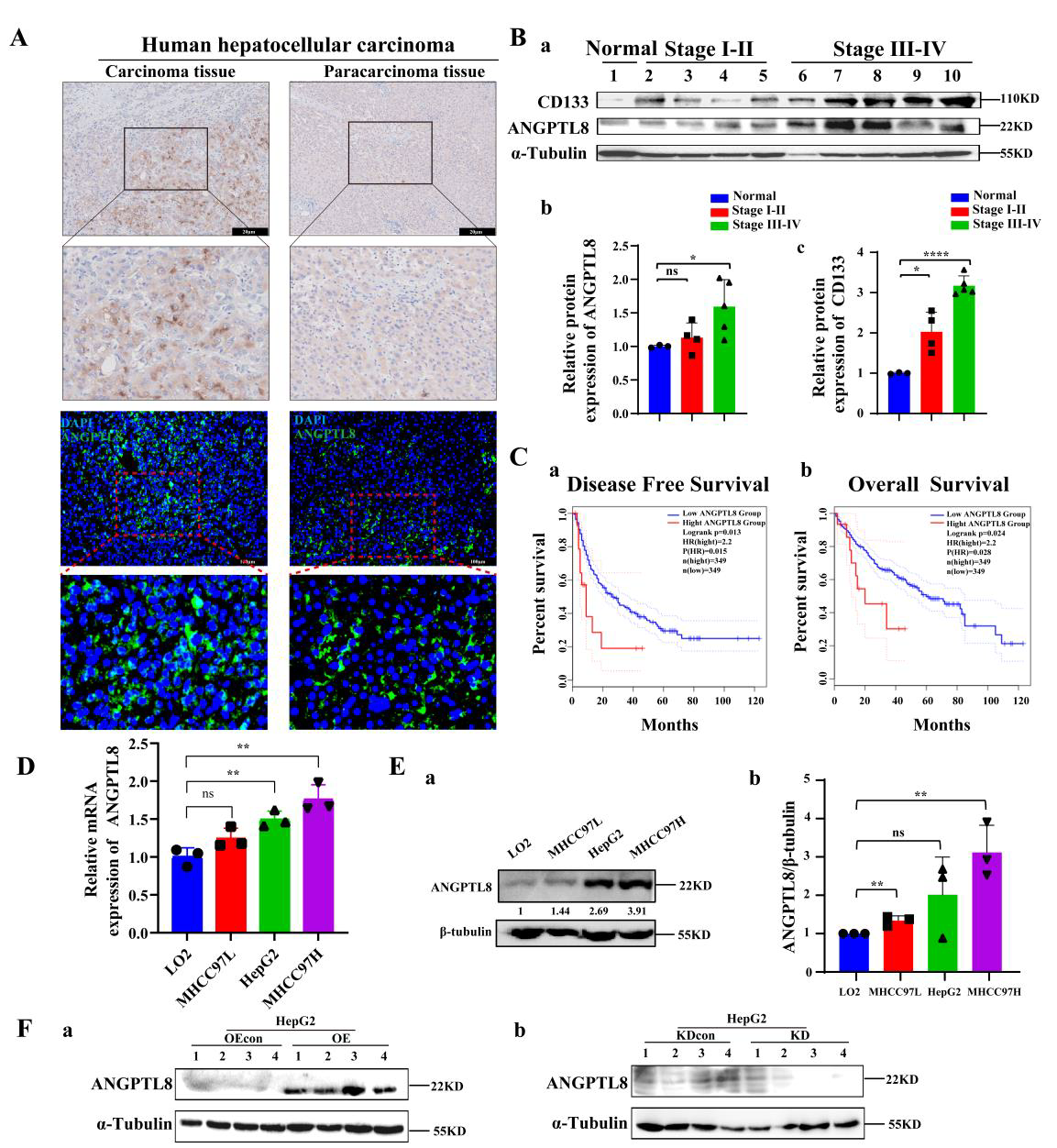


**Supplementary figure legends**

**Supplementary Fig. S1**

**High *ANGPTL8* expression is correlated with malignancy in HCC.**

**A.** IHC and immunofluorescence analysis of ANGPTL8 in human HCC and adjacent paracancerous tissues from thirty individual patients. Scale bars, 20 μm and 100 μm. **B.** (a) Western blot of CD133 and ANGPTL8 protein levels from human HCC of different stages and (b) quantification (n≥3 per group). Data are the mean ± SD. Statistical comparisons were performed using Student’s t test. **p*<0.05, *****p*<0.0001. **C**. Kaplan‒Meier analysis of OS and DFS of patients in the TCGA HCC cohort according to *ANGPTL8* expression level (n=349 per group). **D&E.** Analysis of *ANGPTL8*

6


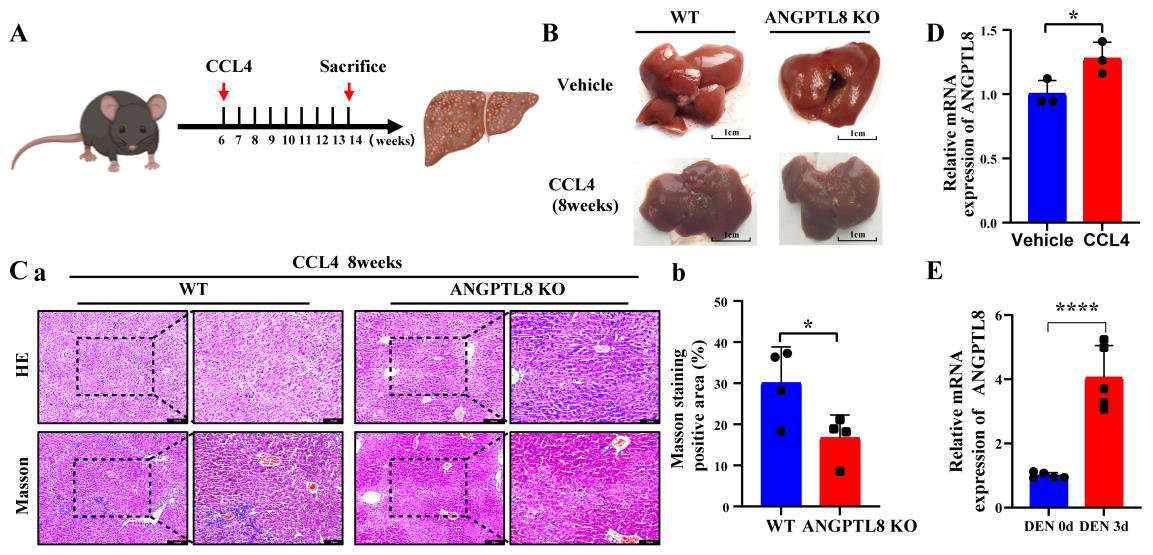


expression in MHCC97H, MHCC97 L and HepG2 HCC cell lines and in the LO2 normal liver cell line by (D) RT‒qPCR and (E) western blotting (n≥3 per group). Protein expression was normalized to β-tubulin. Data are the mean ± SD. Statistical comparisons were performed using Student’s t test. ***p*<0.01. **F.** (a) Analysis of *ANGPTL8* expression in ANGPTL8 OE HepG2 cells, (b) Analysis of *ANGPTL8* expression in ANGPTL8 KD HepG2 cells.

**Supplementary Fig. S2**

***ANGPTL8* knockout alleviates CCL4-induced liver fibrosis.**

**A.** Schematic representing the timeline of CCL4-induced liver fibrosis (n≥8 per group). **B.** Gross appearance of livers from WT and *ANGPTL8*-KO mice induced with CCL4 (n≥8 per group). **C.** (a) Representative images of H&E- and Masson-stained liver tissue from WT and *ANGPTL8*-KO liver fibrosis mouse models and (b) quantification (n=4 per group). Scale bars, 50 μm and 20 μm (inset). Data are the mean ± SD. Statistical comparisons were performed using Student’s t test. **p*<0.05. **D.** mRNA levels of *ANGPTL8* were analyzed by qRT-PCR from the vehicle group and the CCL4 group mouse livers (n=3 per group). **E**. mRNA levels of *ANGPTL8* were determined by qRT- PCR in mouse livers 0 and 3 days after DEN induction (n=5 per group). Data are the mean ± SD. Statistical comparisons were performed using Student’s t test. **p*<0.05, *****p*<0.0001.

7


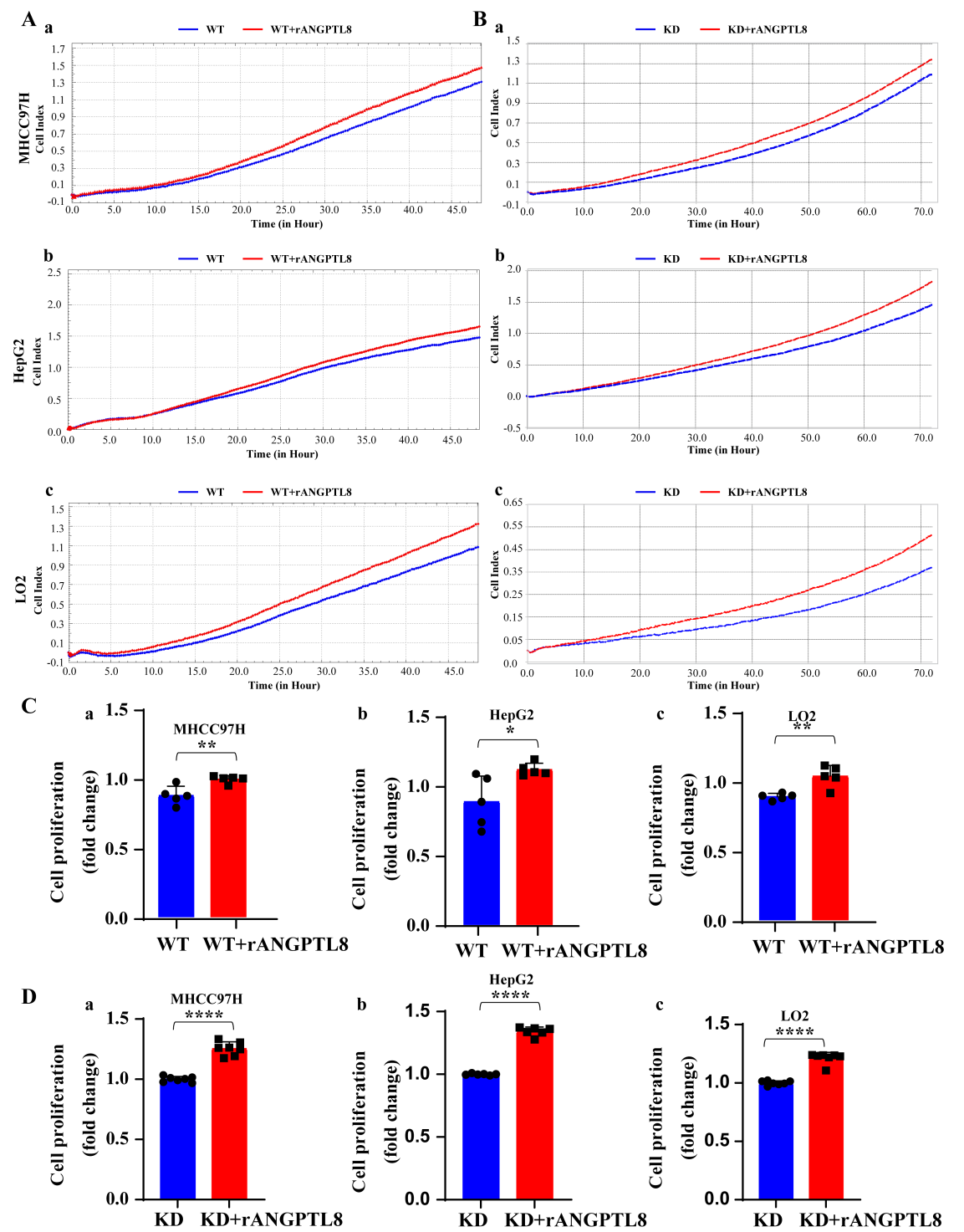


**Supplementary Fig. S3**

**ANGPTL8 promotes normal liver cell and HCC cell proliferation *in vitro*.**

**A.** Detection of the proliferation of (a) MHCC97H, (b) HepG2, and (c) LO2 cells with the addition of human rANGPTL8 (rANGPTL8, 500 ng/ml) to the cell medium using RTCA. **B.** The effect of rANGPTL8 replenishment in ANGPTL8 KD (a) MHCC97H, (b) HepG2, and (c) LO2 cells on cell proliferation was analyzed by RTCA. **C.** Detection

8


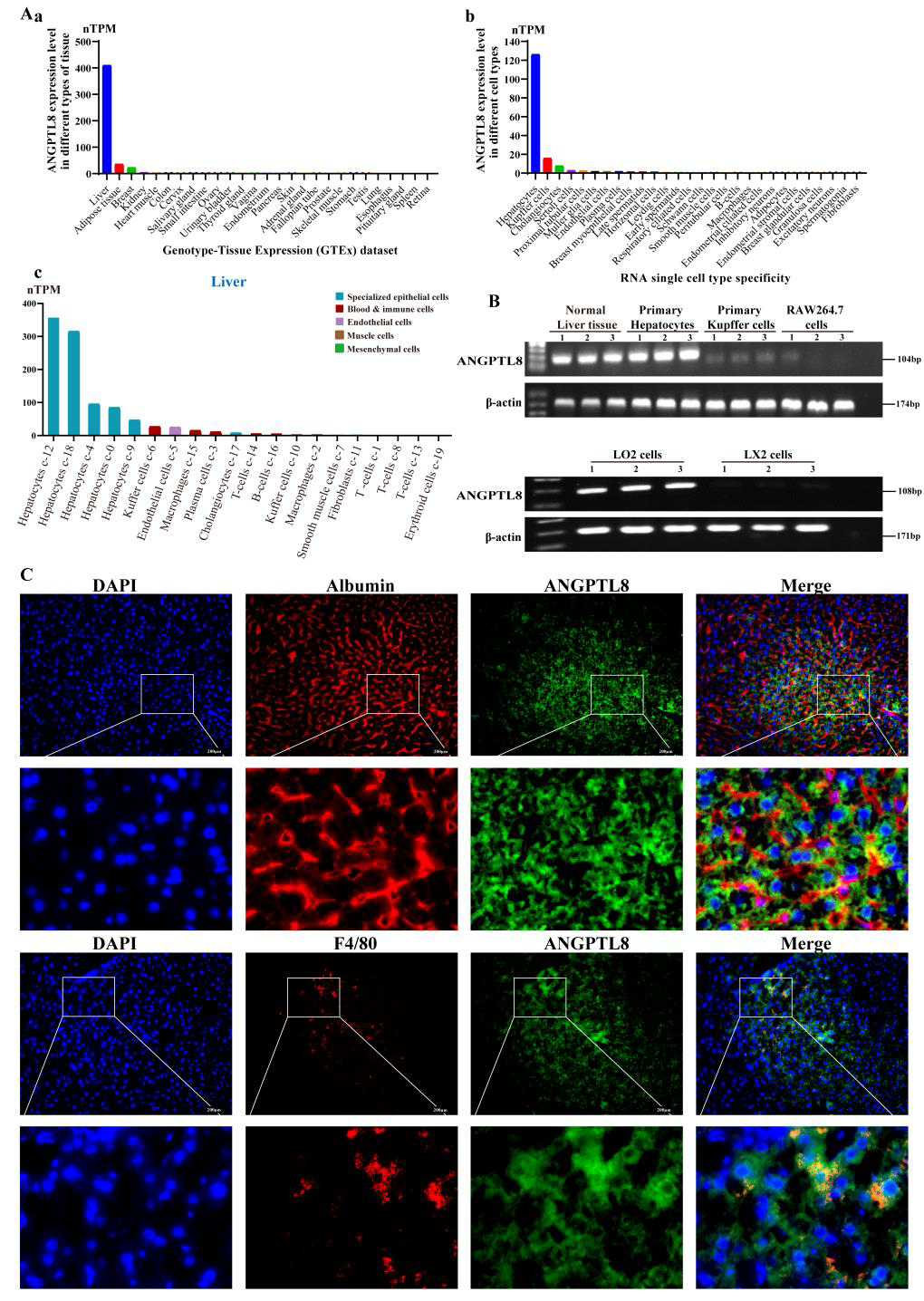


of the proliferation of (a) MHCC97H, (b) HepG2, and (c) LO2 cells with the addition of rANGPTL8 to the cell medium by CCK8 (n=5 per group). **D.** The effect of rANGPTL8 replenishment in ANGPTL8 KD (a) MHCC97H, (b) HepG2, and (c) LO2 cells on cell proliferation was analyzed by RTCA (n≥6 per group). Data are the mean ± SD. Statistical comparisons were performed using Student’s t test. **p*<0.05, ***p*<0.01, *****p*<0.0001.

9

**Supplementary Fig. S4**

**The expression of *ANGPTL8* in different cells.**

**A**. Analysis of the expression of *ANGPTL8* in different tissues and cell types, as well as the different types of cells in the liver by a single-cell database. **B**. Analysis of *ANGPTL8* expression in different cells of the liver and RAW264.7 cells by RT-PCR. **C**. Representative images of immunofluorescence staining of ANGPTL8, Albumin, and F4/80 in the mouse liver tissue.


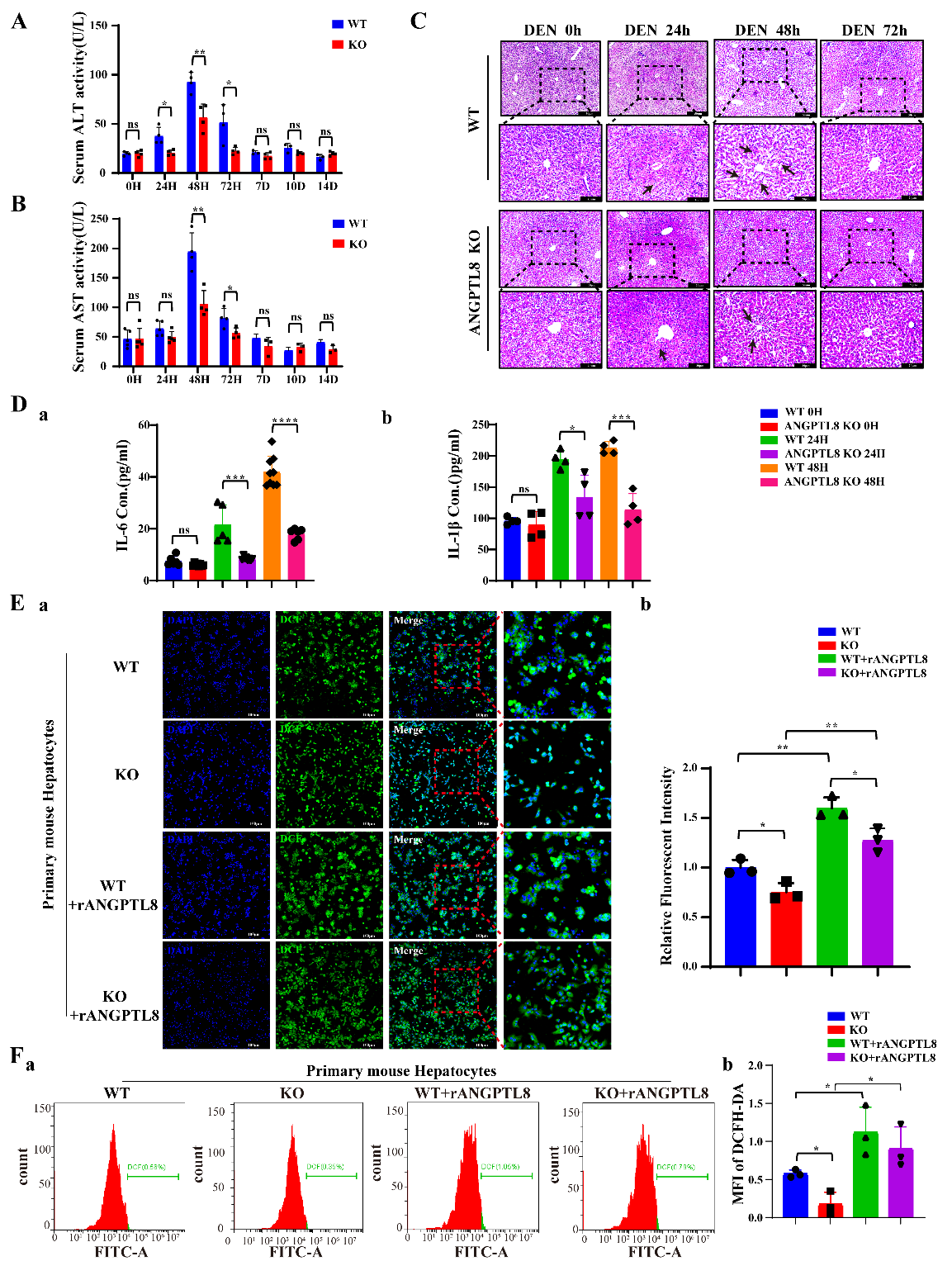


10

**Supplementary Fig. S5**

**ANGPTL8 promotes ROS accumulation during early DEN-induced HCC.**

**A&B.** Serum ALT and AST levels in WT and *ANGPTL8*-KO mice at different time points 24 h, 48 h, 72 h, 7 d, 10 d and 14 d after treatment with DEN (n≥3 per group). Data are the mean ± SD. Statistical comparisons were performed using Student’s t test. **p*<0.05, ***p*<0.01. **C.** Representative H&E staining of liver sections at the indicated time points after DEN treatment. Black arrows indicate necrotic areas. **D.** Serum IL-6 and IL-1β levels were measured by ELISA 24 and 48 hours after DEN treatment (n≥4 per group). Data are the mean ± SD. Statistical comparisons were performed using Student’s t test. **p*<0.05, ****p*<0.001, *****p*<0.0001. **E&F.** Primary hepatocytes were isolated from eight-week-old WT and *ANGPTL8*-KO mice 48 h after DEN administration, and ROS levels were (E) detected with DCFH-DA using a fluorescence confocal microscope and (F) quantified by flow cytometry (n=3 per group). Data are the mean ± SD. Statistical comparisons were performed using Student’s t test. **p*<0.05, ***p*<0.01.

11


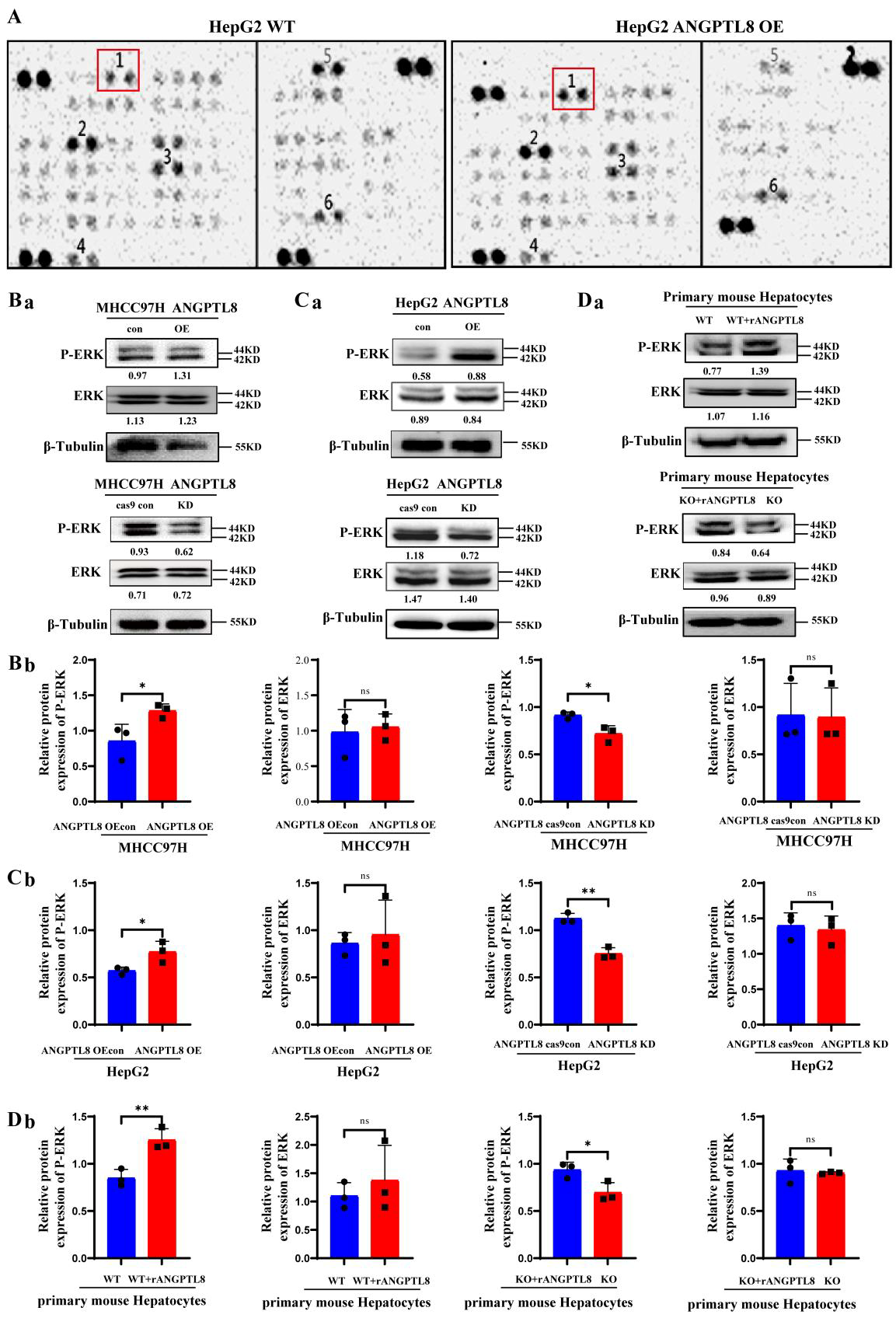


**Supplementary Fig. S6**

**ANGPTL8 upregulates ERK phosphorylation in MHCC97H cells, HepG2 cells, and primary mouse hepatocytes.**

12

**A.** Protein array analysis of the effect of ANGPTL8 on protein phosphorylation in control and *ANGPTL8*-OE HepG2 cells (1: ERK1/2 (T202/Y204); 2: CREB (S133); 3: STAT2 (Y689); 4: PRAS40 (T246); 5: P53 (S392); 6: WNK1 (T60)). **B-D.** Protein levels of total ERK (ERK) and phosphorylated ERK (P-ERK) in (B) MHCC97H cells with *ANGPTL8* OE or knockdown, (C) HepG2 cells with *ANGPTL8* OE or knockdown, and (D) primary mouse hepatocytes isolated from WT and *ANGPTL8*-KO mice. Data are the mean ± SD. Statistical comparisons were performed using Student’s t test. **p*<0.05, ***p*<0.01.

13


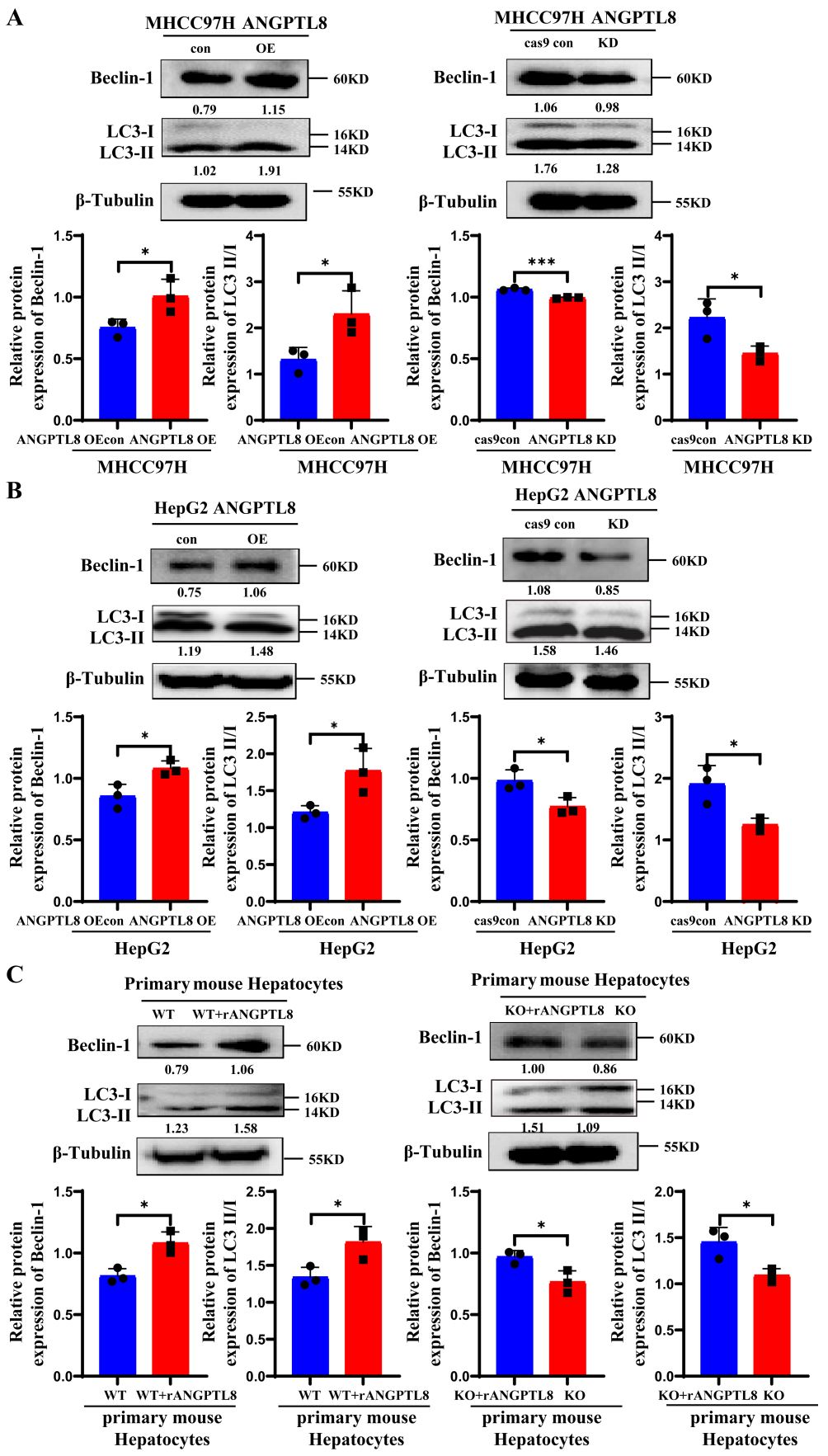


**Supplementary Fig. S7**

**ANGPTL8 regulates autophagy in MHCC97H cells, HepG2 cells with *ANGPTL8***

14


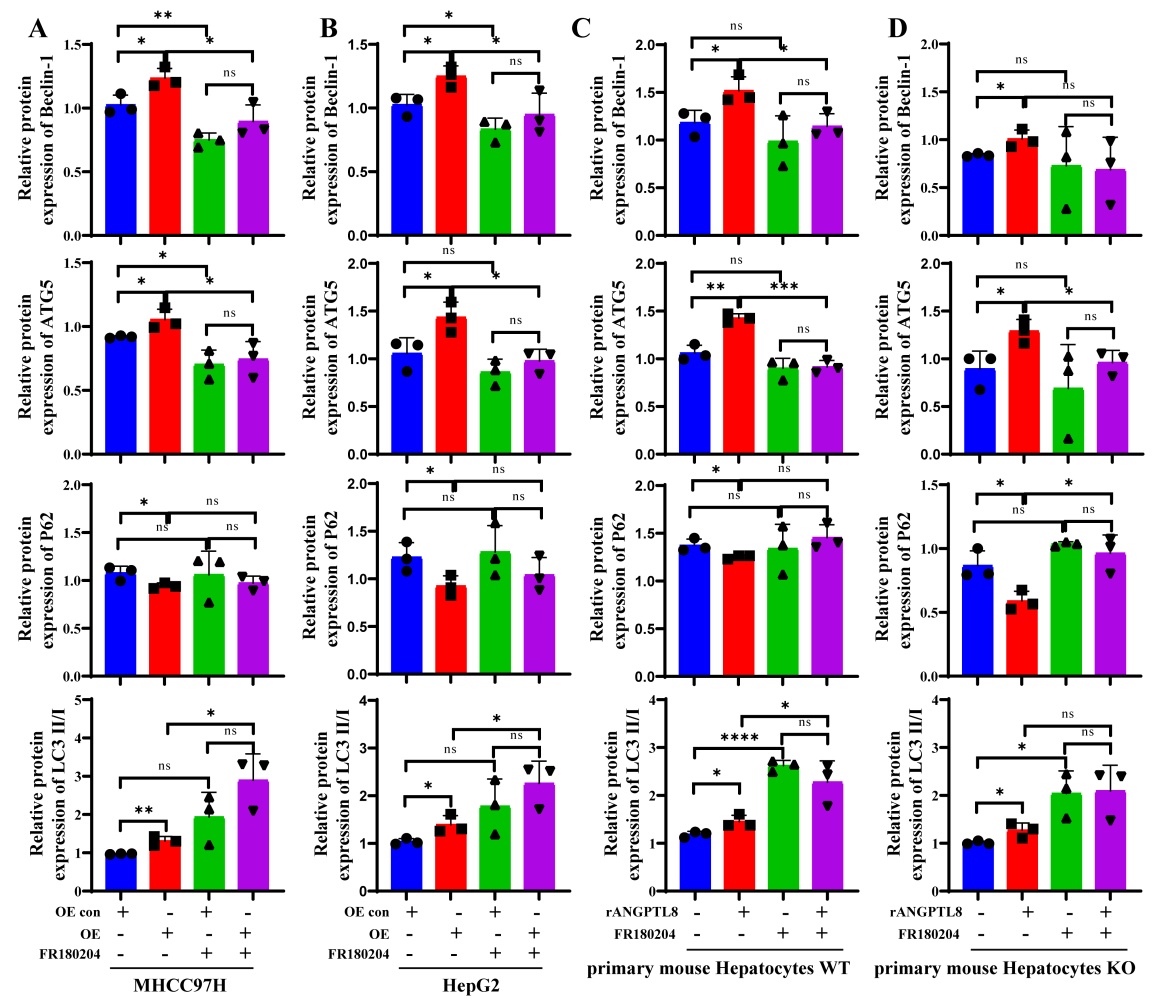


**OE or knockdown and primary mouse hepatocytes cultured with rANGPTL8 protein.**

**A-C.** Beclin-1 and LC3II/I in (A) MHCC97H cells with *ANGPTL8* OE or knockdown, (B) HepG2 cells with *ANGPTL8* OE or knockdown, and (C) WT and *ANGPTL8*-KO primary mouse hepatocytes cultured with rANGPTL*8* protein. Data are the mean ± SD. Statistical comparisons were performed using Student’s t test. **p*<0.05, ****p*<0.001.

**Supplementary Fig. S8**

**ANGPTL8 activates ERK pathway-mediated autophagy.**

**A&B**. Beclin-1, ATG5, P62, and LC3II/I levels in ANGPTL8-overexpressing (A) MHCC97H and (B) HepG2 cells treated with or without the ERK inhibitor FR180204 were detected by western blotting. Protein expression was normalized to β-tubulin or GAPDH, and the numbers represent the mean ± SD of the average of 3 independent experiments. Statistical comparisons were performed using Student’s t test. **p*<0.05,

15

***p*<0.01. **C&D**. Beclin-1, ATG5, P62, and LC3II/I in (C) WT and (D) ANGPTL8- KO primary mouse hepatocytes cultured with or without rANGPTL8 and FR180204 were detected by western blotting. Protein expression was normalized to β-tubulin or GAPDH, and the numbers represent the mean ± SD of an average of 3 independent experiments. Statistical comparisons were performed using Student’s t test. **p*<0.05, ***p*<0.01, ****p*<0.001, *****p*<0.0001.

16


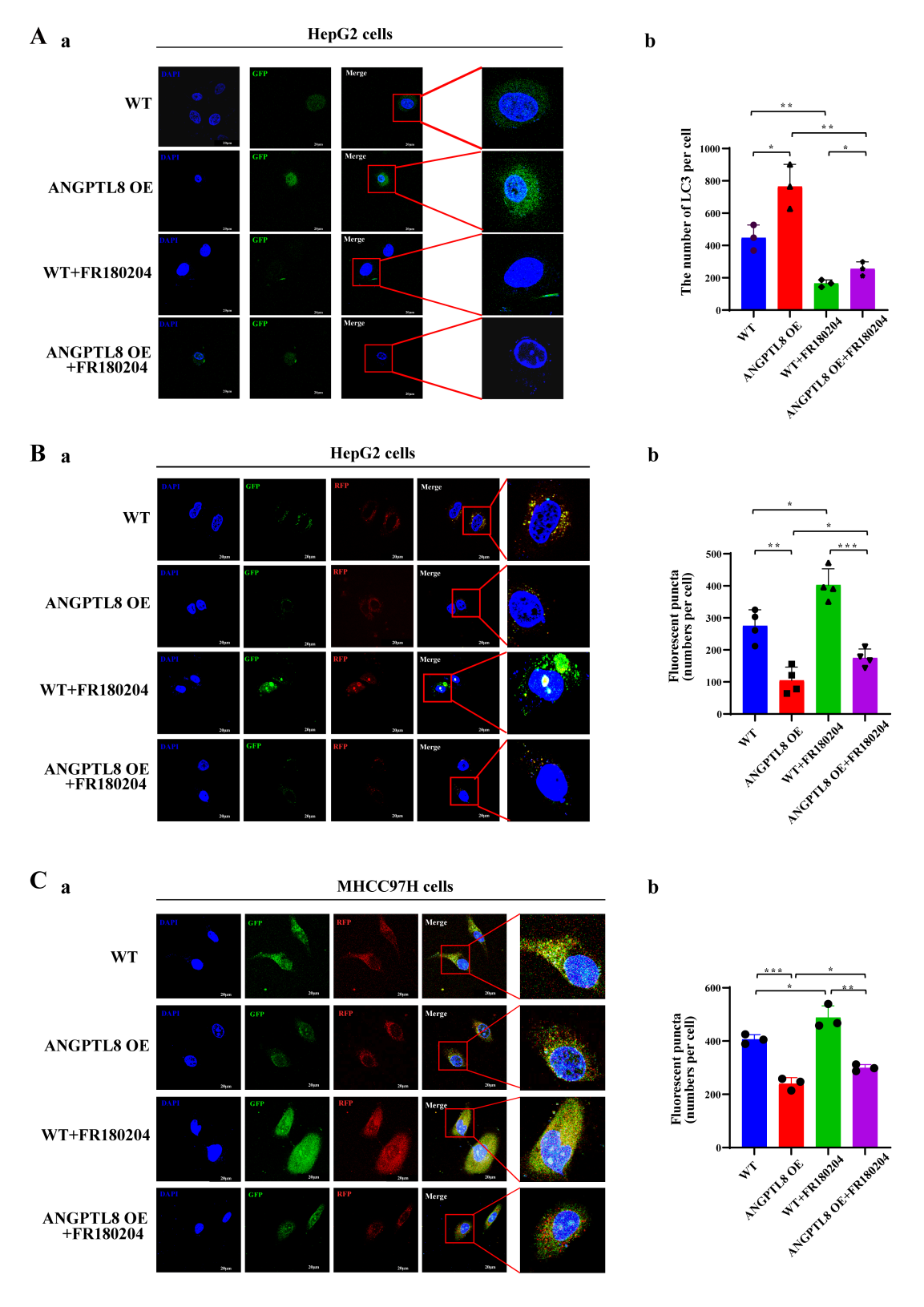


**Supplementary Fig. S9**

**Autophagy levels in HepG2 and MHCC97H cells.**

**A.** Autophagy levels were monitored in HepG2 cells harboring Ad-GFP-LC3B and treated with rANGPTL8 or the ERK inhibitor FR180204. The autophagy level was

17


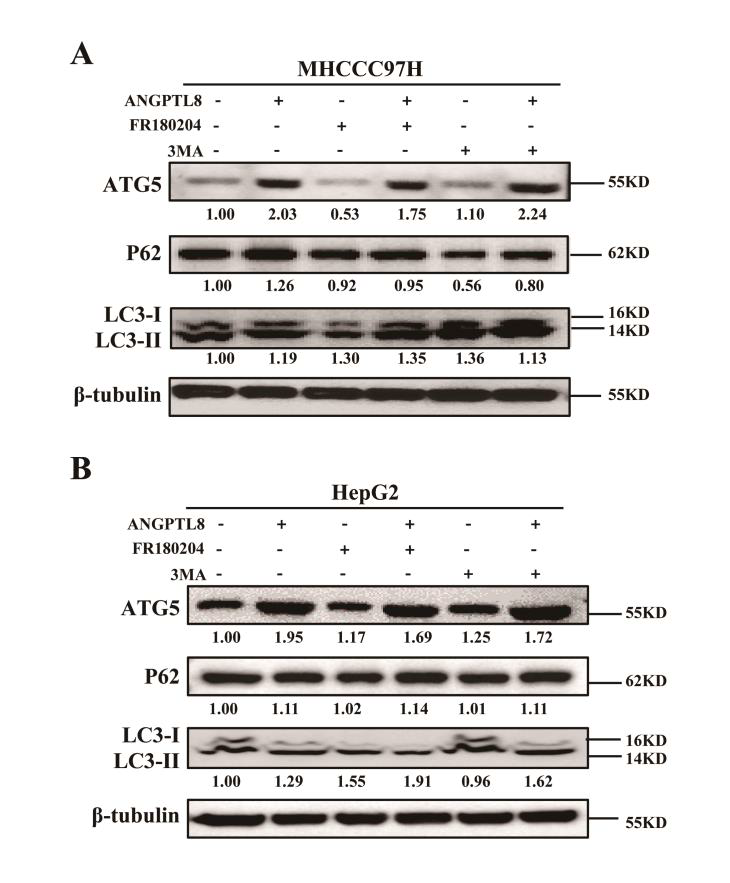


quantified by counting the number of green dots. Data are the mean ± SD. Statistical comparisons were performed using Student’s t test. **p*<0.05, ***p*<0.01. **B.** Autophagic flux was monitored in HepG2 cells harboring Adplus-mCherry-GFP-LC3B and treated with rANGPTL8 or the ERK inhibitor FR180204. Autophagic flux was quantified by counting the number of red and yellow dots. Data are the mean ± SD. Statistical comparisons were performed using Student’s t test. **p*<0.05, ***p*<0.01, ****p*<0.001. **C.** Autophagic flux was monitored in MHCC97H cells harboring Adplus-mCherry- GFP-LC3B and treated with rANGPTL8 or the ERK inhibitor FR180204. Autophagic flux was quantified by counting the number of red and yellow dots. Data are the mean ± SD. Statistical comparisons were performed using Student’s t test. **p*<0.05, ***p*<0.01, ****p*<0.001.

**Supplementary Fig. S10**

**Autophagy-related protein expression in HepG2 and MHCC97H cells.**

**A.** LC3II/I, ATG5 and P62 were detected in *ANGPTL8*-OE MHCC97H cells treated with the ERK inhibitor FR180204 or the autophagy inhibitor 3MA by western blotting.

18


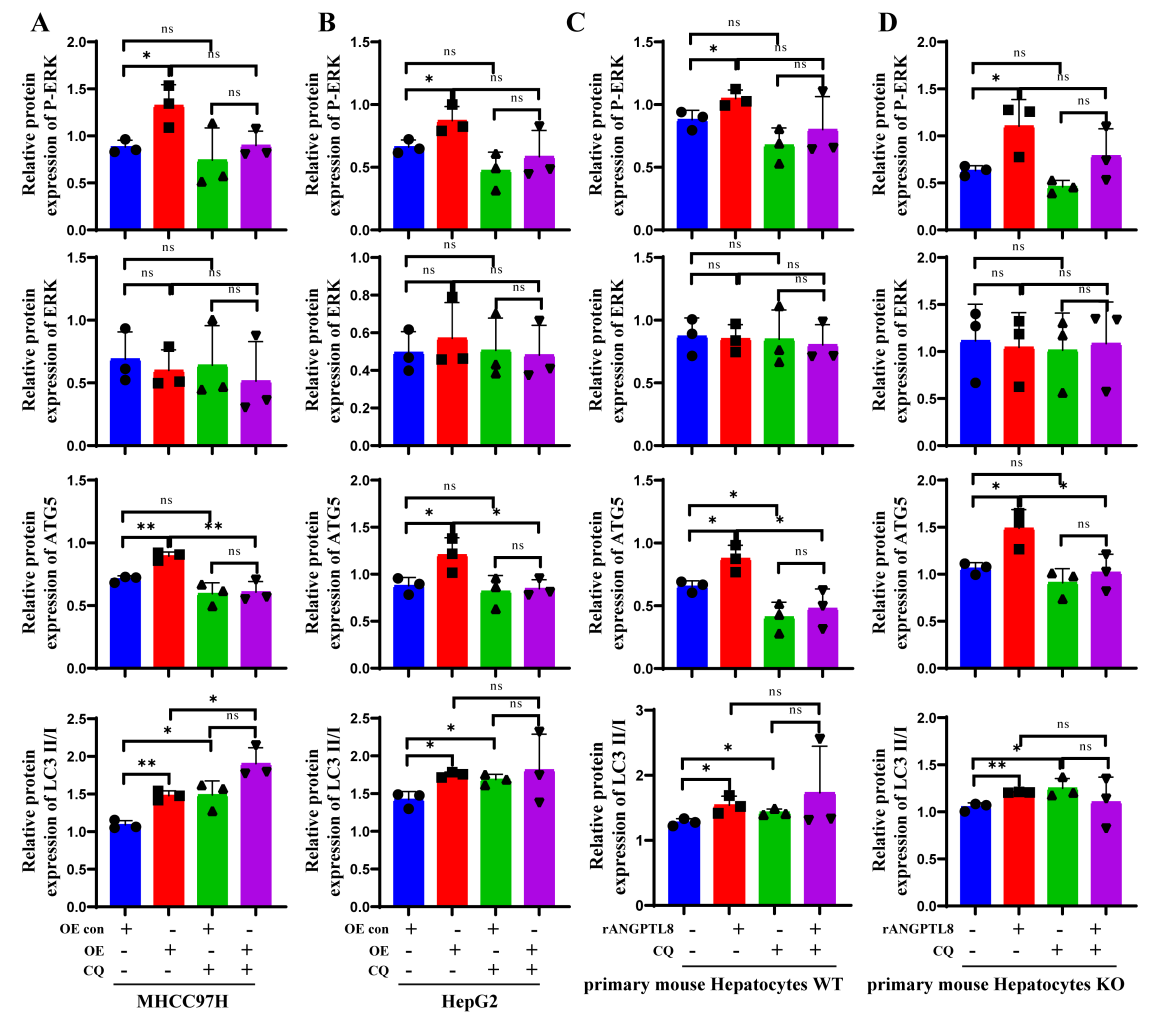


**B.** LC3II/I, ATG5 and P62 were detected in *ANGPTL8*-OE HepG2 cells treated with the ERK inhibitor FR180204 or the autophagy inhibitor 3MA by western blotting.

**Supplementary Fig. S11**

**The activation of autophagy by ANGPTL8 occurs in the late stage of autophagy. A&B**. P-ERK, ERK, ATG5, and LC3II/I levels in ANGPTL8-overexpressing (A) MHCC97H and (B) HepG2 cells treated with or without the autophagy inhibitor CQ were detected by western blotting. Protein expression was normalized to β-tubulin or GAPDH, and the numbers represent the mean ± SD of the average of 3 independent experiments. Statistical comparisons were performed using Student’s t test. **p*<0.05, ***p*<0.01. **C&D**. P-ERK, ERK, ATG5, and LC3II/I levels in (C) WT and (D) ANGPTL8-KO primary mouse hepatocytes cultured with or without rANGPTL8 and autophagy inhibitor CQ were detected by western blotting. Protein expression was normalized to β-tubulin, and the numbers represent the mean ± SD of an average of 3

19


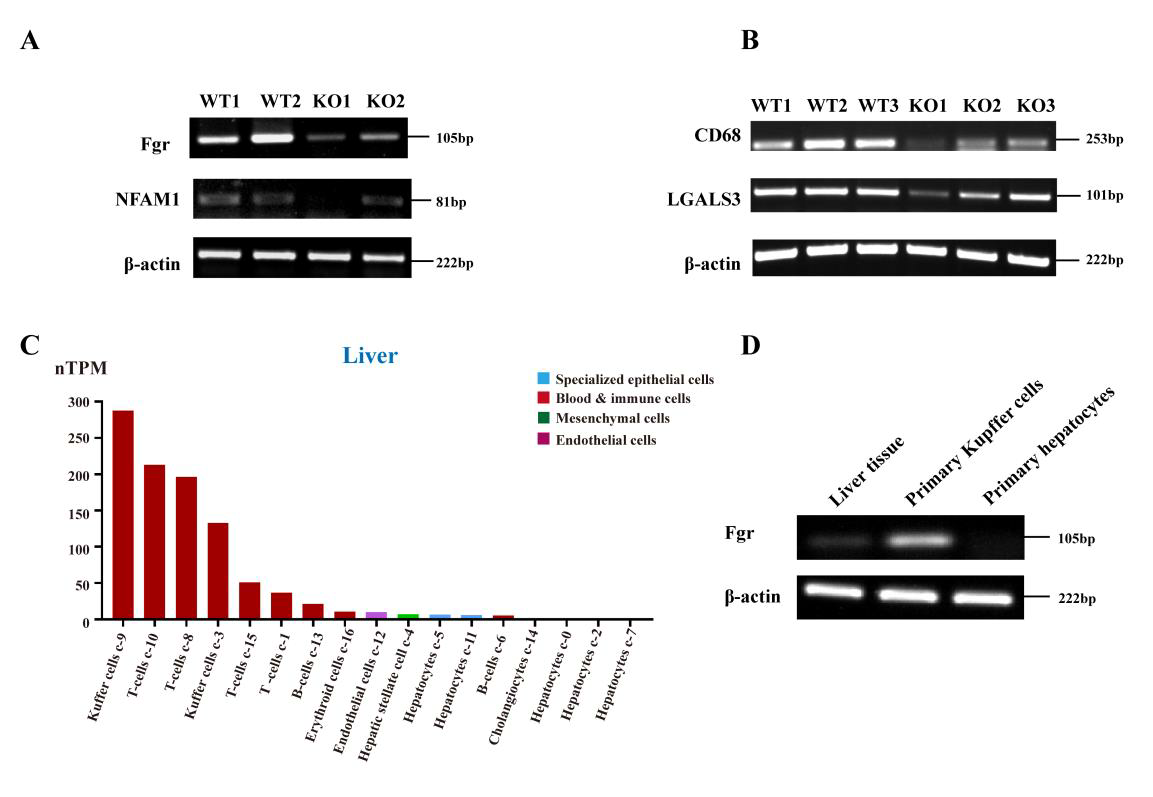


independent experiments. Statistical comparisons were performed using Student’s t test. **p*<0.05, ***p*<0.01.

**Supplementary Fig. S12**

***Fgr* expression in different cells.**

**A&B.** qRT-PCR validation of RNA-seq data for differential gene expression. **C.** *Fgr* expression in different liver cells based on the single-cell database. **D.** qRT-PCR analysis of *Fgr* expression in different cells of the liver.

20


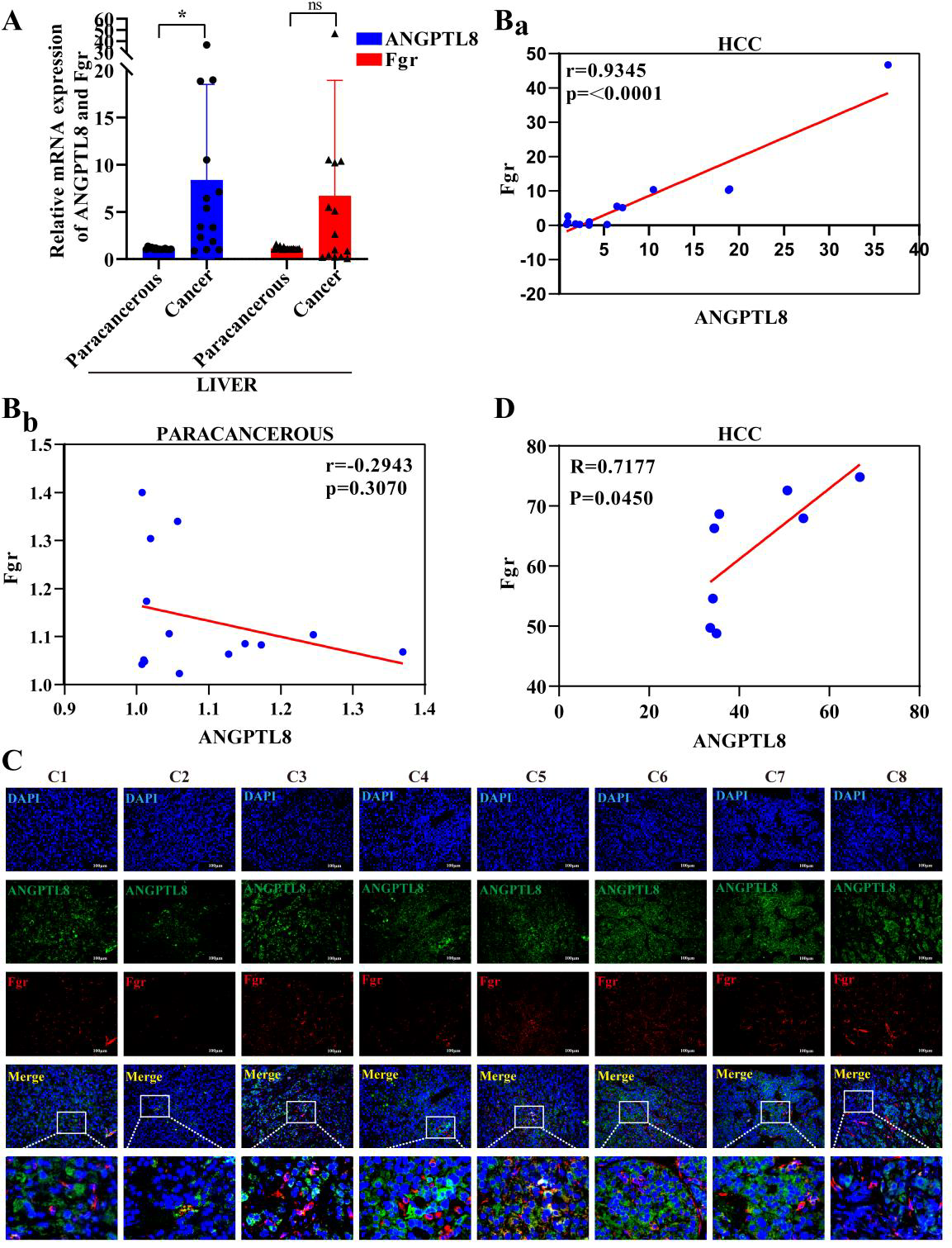


**Supplementary Fig. S13**

**Correlation analysis between ANGPTL8 and Fgr.**

**A.** Analysis of ANGPTL8 and Fgr expression in human HCC and adjacent paracancerous tissues from fourteen individual patients by qRT-PCR. Data are the mean ± SD. Statistical comparisons were performed using Student’s t test. **p*<0.05. **B.** Spearman’s correlation analysis evaluated the association between ANGPTL8 and Fgr in human (a) HCC and (b) adjacent paracancerous tissues from fourteen individual

21


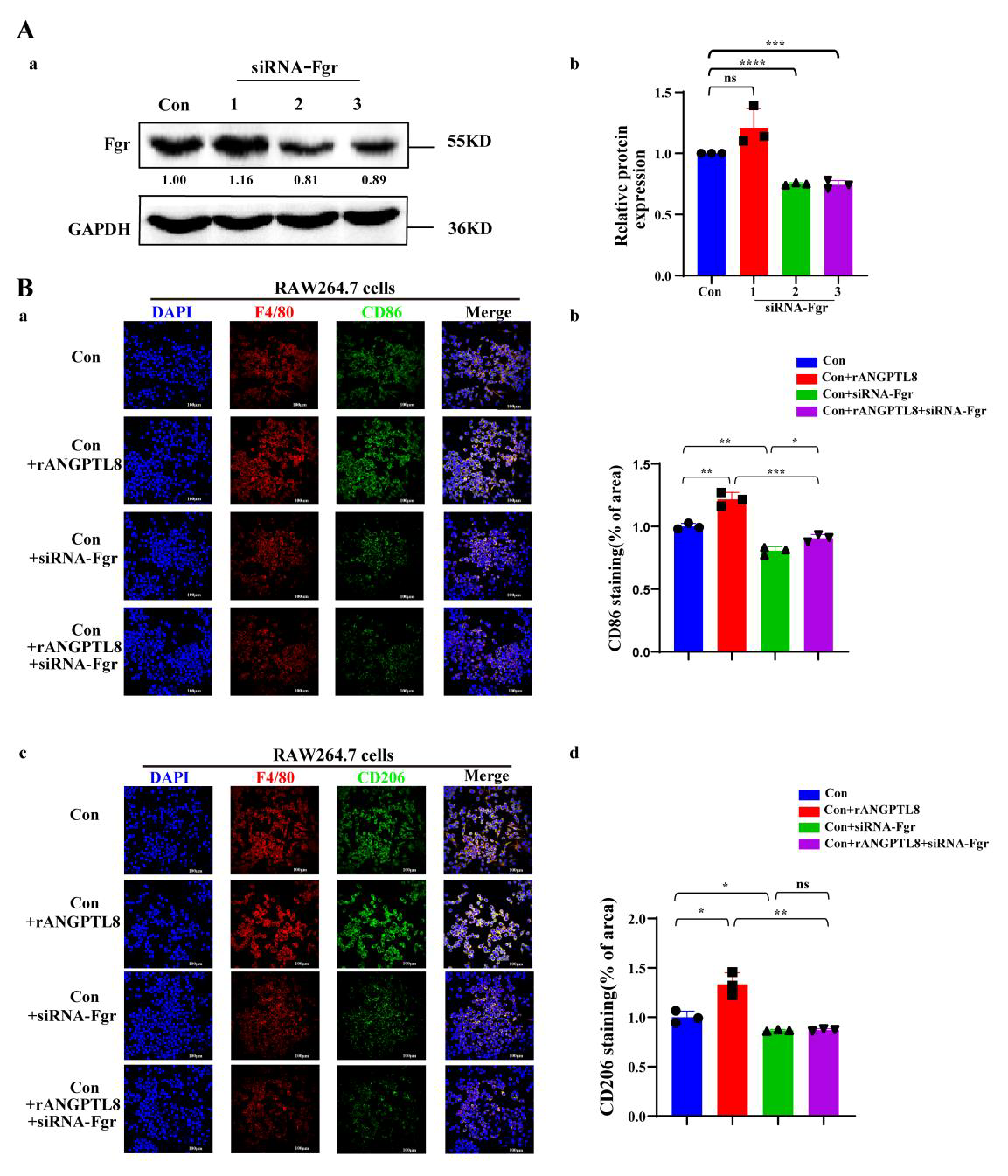


patients by qRT-PCR. **C.** Immunofluorescence analysis of ANGPTL8 and Fgr in human HCC tissues from eight individual patients. Scale bar, 100 μm. **D.** Spearman’s correlation analysis evaluated the association between ANGPTL8 and Fgr in human HCC tissues from eight individual patients.

**Supplementary Fig. S14**

***Fgr* regulated macrophage polarization.**

**A.** *Fgr* expression in RAW264.7 cells transfected with siRNA-1, siRNA-2, and siRNA-

22


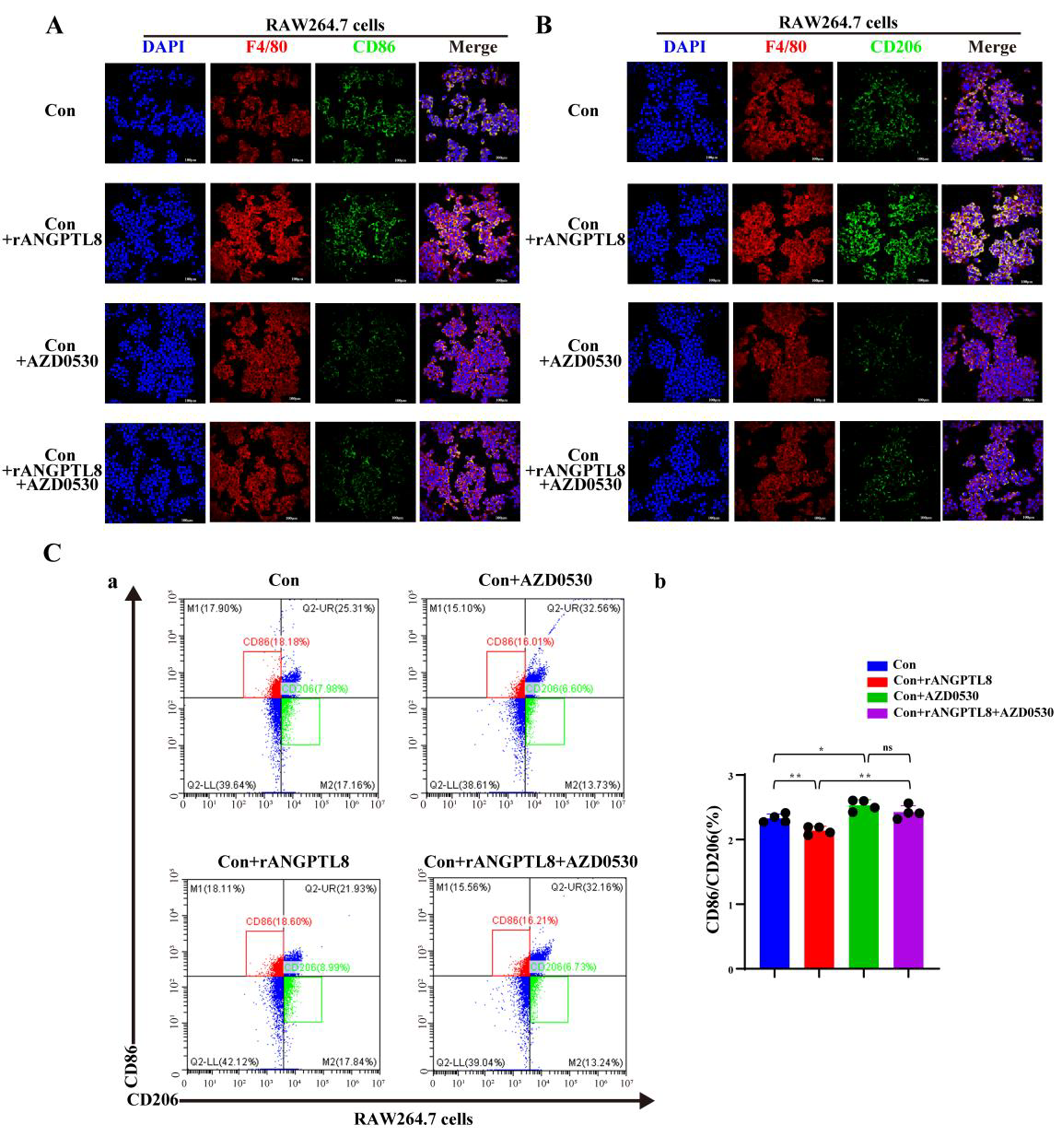


3 compared to the control (Con) (siRNA-GFP) group detected by western blotting. Protein expression was normalized to GAPDH. Data are the mean ± SD. Statistical comparisons were performed using Student’s t test. ****p*<0.001, *****p*<0.0001. **B.** Representative images of cellular immunofluorescence staining of F4/80 and (a&b) CD86 and (c&d) CD206 in RAW264.7 cells with or without rANGPTL8 and *Fgr* siRNA (n=3 per group). Scale bar, 100 μm. Data are the mean ± SD. Statistical comparisons were performed using Student’s t test. **p*<0.05, ***p*<0.01, ****p*<0.001.

**Supplementary Fig. S15**

**Effects of *Fgr* on macrophage polarization.**

23


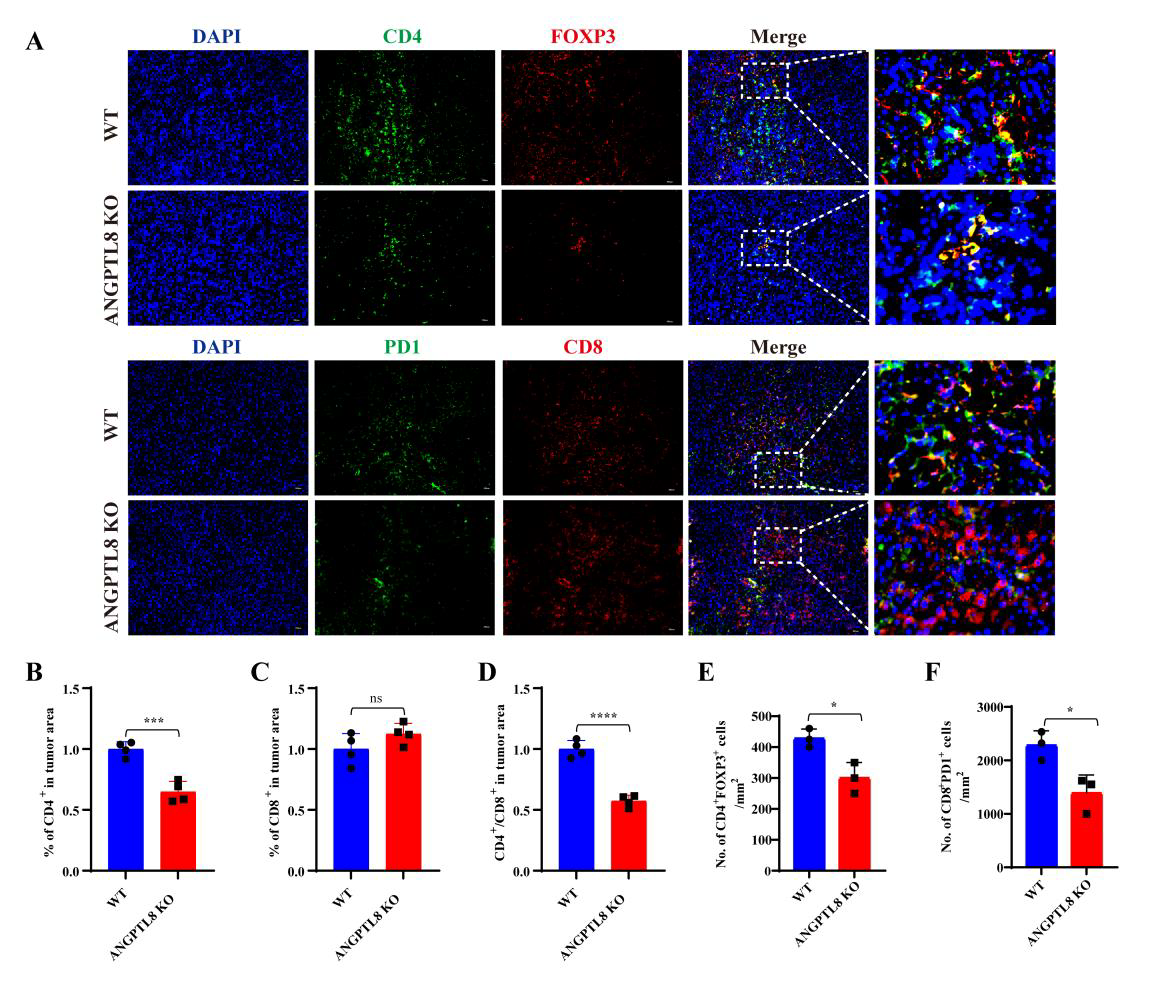


**A**. Representative cellular immunofluorescence image of F4/80 and CD86 in RAW264.7 cells with or without the *Fgr* inhibitor AZD0530. Scale bar, 100 μm. **B.** Representative cellular immunofluorescence images of F4/80 and CD206 in RAW264.7 cells with or without the *Fgr* inhibitor AZD0530. Scale bar, 100 μm. **C.** (a) Flow cytometry analysis of CD86 and CD206 in RAW264.7 cells with or without rANGPTL8 and the *Fgr* inhibitor AZD0530; (b) Statistical analysis of the ratio of CD86/CD206 using Student’s t test. Data are the mean ± SD. **p*<0.05, ***p*<0.01.

**Supplementary Fig. S16**

**ANGPTL8 regulates the number of immunosuppressive CD4^+^FOXP3^+^ and CD8^+^PD1^+^ T cells.**

**A**. Representative immunofluorescence staining image of CD4 and FOXP3, CD8 and PD1 in DEN-induced liver tumor tissue of mice. Scale bar, 200 μm. **B-F.** Statistical analysis of the ratio of CD4^+^FOXP3^+^ and CD8^+^PD1^+^ T cells using Student’s t test. Data

24


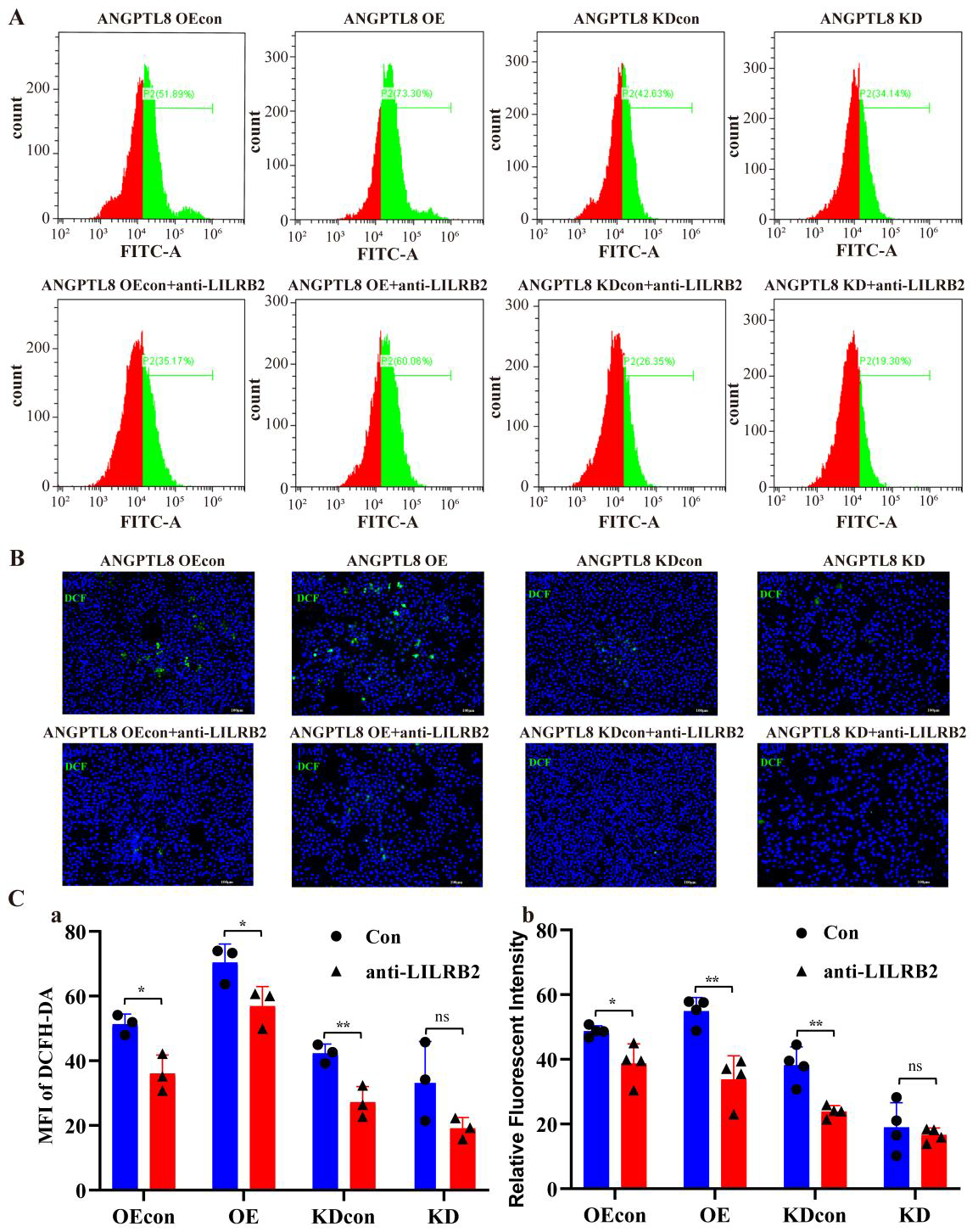


are the mean ± SD. **p*<0.05, ****p*<0.001, *****p*<0.0001.

**Supplementary Fig. S17**

**Blockade of LILRB2 inhibits ANGPTL8-mediated ROS accumulation.**

**A**. ROS levels were detected by flow cytometry in ANGPTL8 OE or KD MHCC97H cells after LILRB2 blockade with an antibody. **B**. ROS levels were detected with DCFH-DA using a fluorescence confocal microscope in ANGPTL8 OE or KD

25


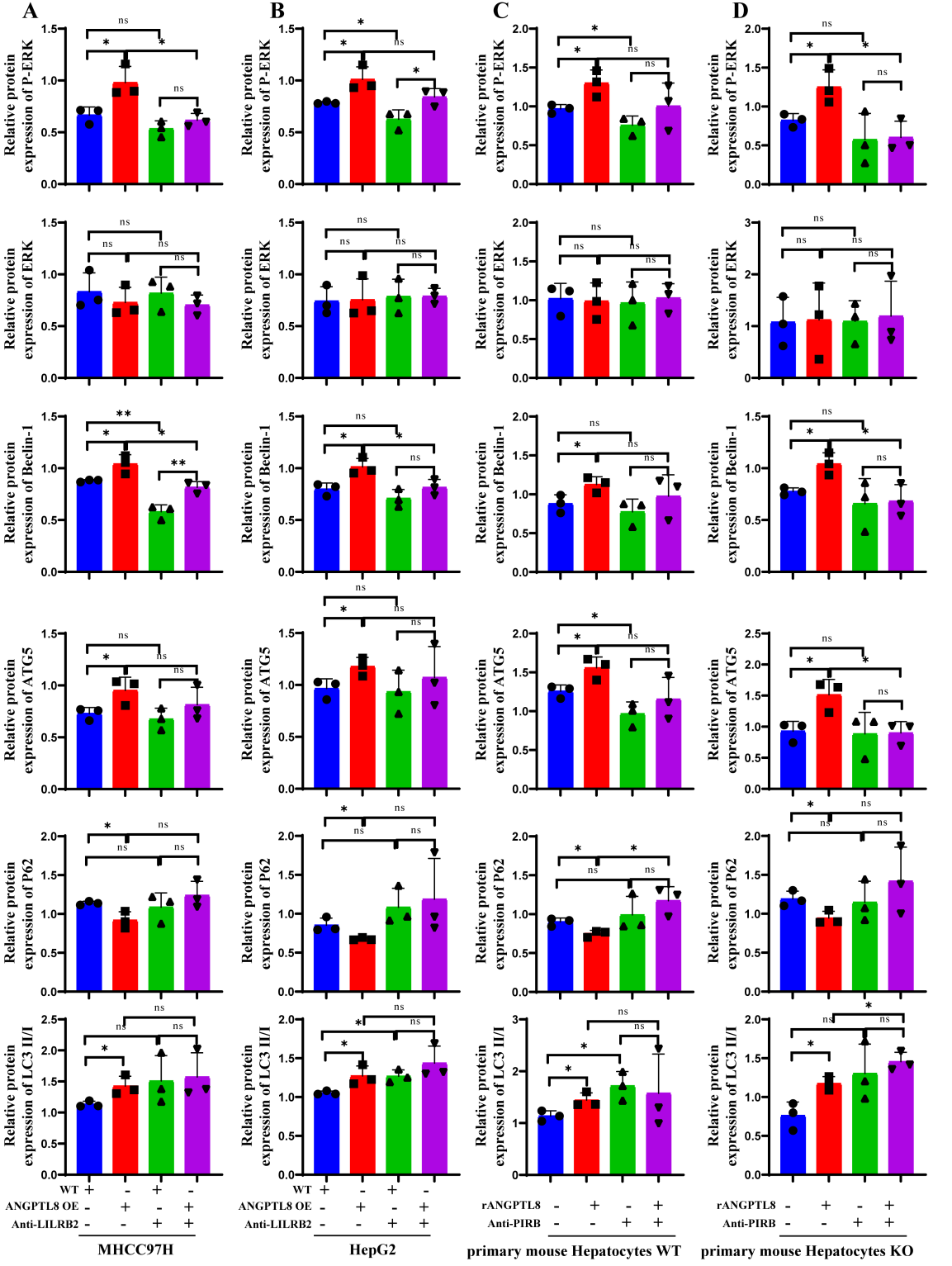


MHCC97H cells after LILRB2 blockade with an antibody. **C.** Statistical analysis of the ratio of flow cytometry (n=3 per group, **p*<0.05, ***p*<0.01) and confocal microscopy (n=4 per group, **p*<0.05, ***p*<0.01). Data are the mean ± SD. Statistical comparisons were performed using Student’s t test.

**Supplementary Fig. S18**

26

**The activation of autophagy by ANGPTL8 occurs in the late stage of autophagy. A&B**. P-ERK, ERK, Beclin-1, ATG5, P62, and LC3II/I levels in ANGPTL8- overexpressing (A) MHCC97H and (B) HepG2 cells treated with or without blocking using an anti-LILRB2 antibody were detected by western blotting. Protein expression was normalized to β-tubulin or β-actin, and the numbers represent the mean ± SD of the average of 3 independent experiments. Statistical comparisons were performed using Student’s t test. **p*<0.05, ***p*<0.01. **C&D**. P-ERK, ERK, Beclin-1, ATG5, P62, and LC3II/I in (C) WT and (D) ANGPTL8-KO primary mouse hepatocytes cultured with or without rANGPTL8 and anti-PIRB antibody were detected by western blotting. Protein expression was normalized to β-tubulin, and the numbers represent the mean ± SD of an average of 3 independent experiments. Statistical comparisons were performed using Student’s t test. **p*<0.05.

27

**Supplementary Table S1.**

**The primer sequences were used for sgRNA to construct the ANGPTL8-CRISPR-** **Cas9 vector**

| **Gene name** | **Primer sequence** |
| --- | --- |
| hbeta-F1(cas9) | CACCGTGGTCCTGTACACACCGTTG |
| hbeta-R1(cas9) | AAACCAACGGTGTGTACAGGACCAC |
| hbeta-F2(cas9) | CACCGGTCCTGTACACACCGTTGA |
| hbeta-R2(cas9) | AAACTCAACGGTGTGTACAGGACC |
| hbeta-F3(cas9) | CACCGCCAGTTCTGGGCCGCCCAT |
| hbeta-Fcas9 | GACCCTCAGTCATGCCAGTG |
| hbeta-Rcas9 | AGAATATCCTCCTCCATCTGCTTA |
| hbeta-F(XhoI peptide-free) | CCGGAATTCATGGCGGCCCCCATG |
| hbeta-F(EcoRI peptide-free) | CCGCTCGAGGGCTGGGAGCGCCGC |

28

**Supplementary Table S2. All the antibodies were used in this study**

| **Experiment** | **Antibody** | **Manufacturer** | **Catalog Number** | **Concentration** |
| --- | --- | --- | --- | --- |
| **IHC** | ANGPTL8 | Abcam | ab180915 | 1:100 |
|  | PCNA | Affinity | AF0239 | 1:200 |
|  | CK18 | Proteintech | 10830-1-AP | 1:200 |
|  | AFP | Absin | abs135527 | 1:200 |
|  | Fgr | Absin | abs115957 | 1:200 |
|  | F4/80 | Affinity | DF2789 | 1:100 |
|  | IL-1β | CST | 12242S | 1:200 |
| **Western**  **blot** | ANGPTL8 | Abcam | ab180915 | 1:1000 |
|  | CD133 | Abcam | ab222782 | 1:1000 |
|  | Beclin-1 | CST | 3495S | 1:1000 |
|  | ATG5 | Beyotime | AF2269 | 1:1000 |
|  | P62 | CST | 5114s | 1:1000 |
|  | LC3I/II | Proteintech | 14600-1-AP | 1:1000 |
|  | ERK1/2 | Beyotime | AF1051 | 1:1000 |
|  | p-ERK1/2 | CST | 4370s | 1:1000 |
|  | LILRB2 | ABclonal | A10135 | 1:500 |
|  | α-Tubulin | Beyotime | AF2827 | 1:1000 |
|  | β-tubulin | Beyotime | AF2835 | 1:1000 |
| **FCM** | CD16/32 | BioLegend | 156603 | 1:100 |
|  | CD45 | BioLegend | 103105 | 1:100 |
|  | CD11b | BioLegend | 101225 | 1:100 |
|  | F4/80 | BioLegend | 123107 | 1:50 |
|  | CD86 | BioLegend | 105011 | 1:100 |
|  | CD206 | BioLegend | 141715 | 1:100 |

29

| **IF** | Albumin | Proteintech | 66051-1-Ig | 1:200 |
| --- | --- | --- | --- | --- |
|  | F4/80 | Abcam | ab6640 | 1:50 |
|  | Fgr | Absin | abs115957 | 1:100 |
|  | CD86 | Proteintech | 13395-1-AP | 1:100 |
|  | CD206 | Proteintech | 18704-1-AP | 1:100 |
|  | FOXP3 | ThermoFisher | 14-4776-82 | 1:200 |
|  | CD4 | Abcam | ab183685 | 1:100 |
|  | CD8 | Santa Cruz | sc-7970 | 1:100 |
|  | PD1 | Abcam | ab214421 | 1:200 |
|  | ANGPTL8 | Abcam | ab180915 | 1:50 |

30

**Supplementary Table S3.**

**The primer sequences of qPCR were used in the experiment**

| **Gene name** | **Primer sequence** | **Amplicon size** |
| --- | --- | --- |
| H-F-β-actin | AAACTGGAACGGTGAAGGTG | 171 bp |
| H-R-β-actin | AGAGAAGTGGGGTGGCTTTT | 171 bp |
| M-F-β-actin | GTGCTATGTTGCTCTAGACTTCG | 174 bp |
| M-R-β-actin | ATGCCACAGGATTCCATACC | 174 bp |
| M-F-β-actin | CTGGAACGGTGAAGGTGACA | 222 bp |
| M-R-β-actin | AAGGGACTTCCTGTAACAACGC | 222 bp |
| H-F-ANGPTL8 | GCCTGTTGGAGACTCAGATGGA | 108 bp |
| H-R-ANGPTL8 | CGCTGTCCCGTAGCACCTTC | 108 bp |
| M-F-ANGPTL8 | CTGCCTCCTGTGGACCTTAG | 104 bp |
| M-R-ANGPTL8 | TCTGTACACGCCATTGAGGG | 104 bp |
| H-F-Fgr | CAGCAACTTCTCCTCTCAG | 115 bp |
| H-R-Fgr | CGAGCCTCATAGTCATACAG | 115 bp |
| M-F-Fgr | TTGGAAAGATTGGGAGAAAG | 105 bp |
| M-R-Fgr | ATGGACAGGGAGTAGGCAC | 105 bp |
| M-F-CD68 | TGGCGGTGGAATACAATG | 253 bp |
| M-R-CD68 | GATGAGAGGCAGCAAGAG | 253 bp |
| M-F-LGALS3 | TGGTTCCAGGGACTCAAGGTA | 101 bp |
| M-R-LGALS3 | CCACCGGCCTCTGTAGAAGA | 101 bp |
| M-F-NFAM1 | AAGCCTCCAGCAGAATCT | 81 bp |
| M-R-NFAM1 | TTCCAGTCTCTTCCTTCATAC | 81 bp |

31
